# Restoring the Immunity in the Tumor Microenvironment: Insights into Immunogenic Cell Death in Onco-Therapies

**DOI:** 10.3390/cancers13112821

**Published:** 2021-06-05

**Authors:** Ángela-Patricia Hernández, Pablo Juanes-Velasco, Alicia Landeira-Viñuela, Halin Bareke, Enrique Montalvillo, Rafael Góngora, Manuel Fuentes

**Affiliations:** 1 Department of Medicine and General Cytometry Service-Nucleus, CIBERONC CB16/12/00400, Cancer Research Centre (IBMCC/CSIC/USAL/IBSAL), 37007 Salamanca, Spain; angytahg@usal.es (Á.-P.H.); pablojuanesvelasco@usal.es (P.J.-V.); alavi29@usal.es (A.L.-V.); halin.bareke@gmail.com (H.B.); emontalvillo@usal.es (E.M.); rgongora@usal.es (R.G.); 2 Department of Pharmaceutical Biotechnology, Faculty of Pharmacy, Institute of Health Sciences, Marmara University, 34722 Istanbul, Turkey; 3 Proteomics Unit, Cancer Research Centre (IBMCC/CSIC/USAL/IBSAL), 37007 Salamanca, Spain

**Keywords:** immunogenic cell death, DAMPs, apoptosis, necroptosis, autophagy, tumor microenvironment, natural products, chemotherapy, ICD inducers, immunotherapy

## Abstract

**Simple Summary:**

Since the role of immune evasion was included as a hallmark in cancer, the idea of cancer as a single cell mass that replicate unlimitedly in isolation was dissolved. In this sense, cancer and tumorigenesis cannot be understood without taking into account the tumor microenvironment (TME) that plays a crucial role in drug resistance. Immune characteristics of TME can determine the success in treatment at the same time that antitumor therapies can reshape the immunity in TME. Here, we collect a variety of onco-therapies that have been demonstrated to induce an interesting immune response accompanying its pharmacological action that is named as “immunogenic cell death”. As this report shows, immunogenic cell death has been gaining importance in antitumor therapy and should be studied in depth as well as taking into account other applications that may arise from this immune phenomenon.

**Abstract:**

Immunogenic cell death (ICD) elicited by cancer therapy reshapes the tumor immune microenvironment. A long-term adaptative immune response can be initiated by modulating cell death by therapeutic approaches. Here, the major hallmarks of ICD, endoplasmic reticulum (ER) stress, and damage-associated molecular patterns (DAMPs) are correlated with ICD inducers used in clinical practice to enhance antitumoral activity by suppressing tumor immune evasion. Approaches to monitoring the ICD triggered by antitumoral therapeutics in the tumor microenvironment (TME) and novel perspective in this immune system strategy are also reviewed to give an overview of the relevance of ICD in cancer treatment.

## 1. Introduction

**Figure 1:**
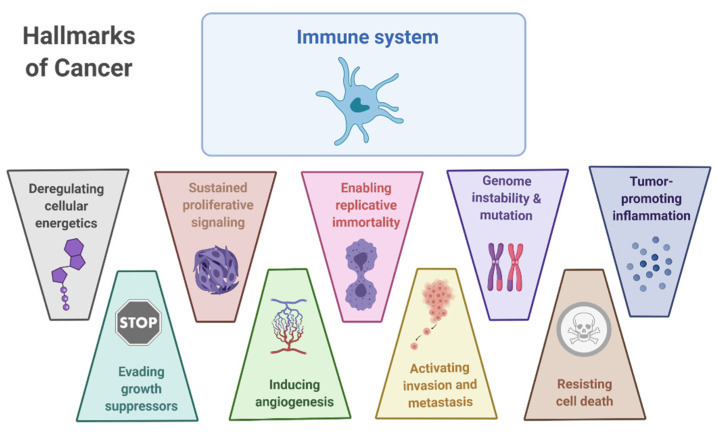
The immune system within the cancer hallmarks plays a pivotal role in understanding of the TME.

Since the establishment of tumorigenesis as a complex network of pathophysiological processes, cancer cell biology cannot be studied without taking into account the surrounding elements (e.g., blood vessels, immune cells, and extracellular matrix). Tumor cells have acquired mechanisms to hinder anti-tumor immunity. The tumor’s ability to evade the immune system is one of the steps that have been considered necessary for tumor establishment and has been included in multi-step processes that have been identified as “hallmarks of cancer” proposed by Hanahan and Weinberg [1]. The role of immune evasion was included as a hallmark when the idea of cancer as a single cell mass that replicates unlimitedly in isolation was dissolved (Figure 1). Rather, it was accepted that a set of normal cells that are recruited to the tumor or that are altered by the tumor to form the tumor-associated stroma, were pivotal for tumor establishment and progression. Therefore, the cancer cell and tumorigenesis cannot be understood without taking into account the tumor microenvironment (TME) [2]. In-depth understanding of molecular signaling and extracellular interactions involved in these processes allows the development of novel therapies that include conventional intracellular targets as well as novel approaches to modulate the TME. 

Treatment resistance remains an important challenge in cancer therapy. Drug resistance can be intrinsic due to the presence of mutations that inactivate its potential molecular targets [3]. However, extrinsic resistance of the tumor cells can be promoted by the tumor surrounding. As stated before, tumors are surrounded by a high density of elements such as extracellular matrix, aberrant vasculature, and stroma cells. Within this cellular component surrounding the tumor, tumor-infiltrating cells are of special interest because of their contradictory roles in immunomodulation of tumor progression. While some cell populations boost tumorigenic factors, others can restore immunosurveillance against the tumors. In TME, the balance is tilted towards the activation of tumor survival-promoting elements that enable tumor immune evasion [4]. Therefore, therapies that target the immunosuppressive environment in the TME has the potential to unlock the power of the immune system against tumor cells.

## 2. Cancer Immunoediting, TME, and Immunogenic Cell Death

Interactions between the immune system and the tumor, even before the onset of the clinical signs of a tumor, are well characterized. This process encompasses different steps that have been termed jointly as immunoediting [5]. Cancer immunoediting defines the interaction between immune cells and tumor cells that can result in either elimination of the tumor, an equilibrium state where the tumor is latent or generation of tumor cell populations that are able to survive in immunocompetent hosts [6]. First, pre-tumoral changes are detected and neutralized by the innate and adaptative immune system in the step named “elimination”. In the “equilibrium” phase, the tumor is not completely eliminated; however, it is kept under control by the immune system so that it doesn’t become clinically detectable. Finally, tumors develop an editing process where immunogenic tumor cells are ‘selected against’ by immunoselection [7]. Therefore, the cell populations that end up establishing the tumors are the ones that can evade the immune recognition and response.

The steps involved in an effective anti-tumor immune response for the elimination of tumors have been described in the cancer immunity cycle [7]. In cancer immunity cycle, dying cells are phagocyted by antigen-presenting cells (APCs) such as dendritic cells (DCs). Also, these cells can capture the released tumor-associated antigens (TAAs). TAAs play a pivotal role in distinguishing tumor tissue from normal cells. Currently, this part of the tumor immune cycle is being considered as the rational basis for new anti-tumor therapies such as vaccines [8,9]. TAAs must then be presented to naive T cells through MHC-I and MHC-II molecules to activate CD8+ T cells and CD4+ T cells, respectively. Finally, T cells are recruited to infiltrate the TME to kill the tumor cells through recognition by the T-cell receptor (TCR) of the MHC-peptide complex [7]. Tumoral cell death and TAAs released complete cancer immunity cycle. Within this complex and meticulous process, there are multiple points where the continuity of the immune response can be altered or lost, leading to tumor evasion. 

**Figure 2:**
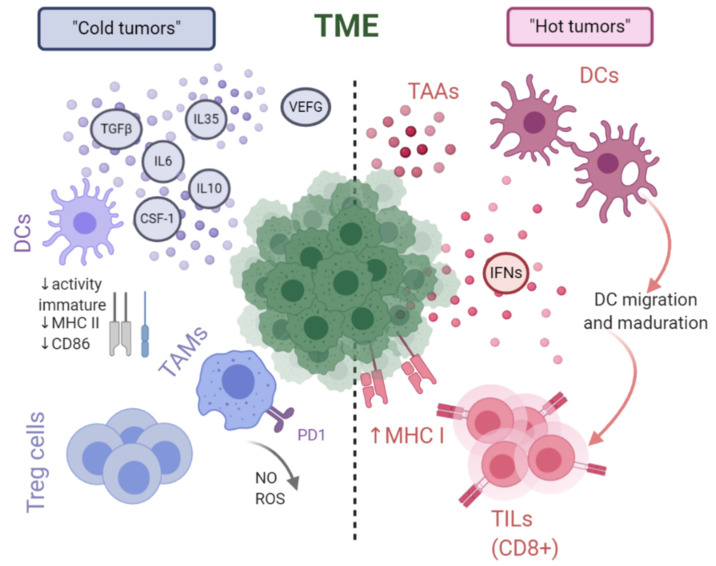
Main cells and signaling components involved in tumor microenvironment (TME) that confers lack (“cold” tumors) or enhancement (“hot” tumors) of immunogenicity to cancer cells.

Considering the immunogenicity of tumors, their intrinsic characteristics and the accompanying TME, tumors can be divided into two types. This distinction derives from the different composition of cytokines, inflammatory agents or cell populations that together create a pro- or anti-tumor environment. The presence of soluble pro-inflammatory factors (e.g., IFNs) or T-cell infiltration of the tumor increases immunogenicity, and these are referred to as “hot tumors”. The opposite situation, “cold tumors”, is characterized by the presence of immunosuppressive lymphoid and myeloid cells such as regulatory T cells or tumor-associated macrophages together with anti-inflammatory cytokines such as IL-6 or IL-10 [10]. The specific characteristics of these two situations are shown in more detail in Figure 2.

Immunotherapy and novel chemotherapy base their successes on the immunogenicity of tumors [11]. On the one hand, it is necessary to increase the recruitment of T cells in the TME. On the other hand, it is also required to promote the entire immune cycle machinery for the process to be successfully completed. In this sense, the key may lie in the modulation of cell death to promote greater tumor immunogenicity. Cell death has been considered a silent immune process as it is the case with regulated cell death (RCD), associated with post-embryonic development and the maintenance of tissue homeostasis. In contrast, pathogenic cell death develops a “danger” signal that stimulates the innate immune response by the release of pathogen-associated molecular patterns (PAMPs) and/or danger-associated molecular patterns (DAMPs). It has been shown that under certain circumstances of stress, cells are able to develop a pro-inflammatory process that culminates in the increase of T cell activation that has been termed “immunogenic cell death” (ICD) [12]. The ICD concept has been identified as a single class of RCD that elicits complete adaptive immune responses through the emission of danger signals (DAMPs). Release of DAMPs, which normally function intracellularly, plays an immunogenic role when they reach the TME. 

After briefly summarizing the main role of the immune system in TME and the relevance of ICD for immunomodulation of tumor surroundings, we will discuss the molecular players and processes that trigger ICD such as endoplasmic reticulum (ER) stress and DAMPs. We will also explore the current ways of targeting TME with ICD, as well as the experimental strategies and perspectives. To this end, a systematic review of the literature on the topic covered in the report has been carried out through the platforms “Web of Science” (https://apps.webofknowledge.com, accessed on 10 January 2021) and PubMed (https://pubmed.ncbi.nlm.nih.gov, accessed on 10 January 2021). For this purpose, the general term “immun has been combined with key terms such as “immunogenic cell death”, “DAMPs” or “TME”. The search has also been performed by combining therapy-related terms such as “anti-tumour”, “onco-” or “drug”. For a more specific search on the molecules with pharmacological activity included in this work, Scifinder (https://scifinder.cas.org, accessed on 10 January 2021) database has also been accessed. This study has not included any filtering by time of publication in order to give a global approach to the trajectory of the topic from the earliest evidence to the most recent trends.

## 3. Cell Death and DAMPs: Restoring Immunity in TME

Cell death classification has expanded over the last years, and the introduction of “immunogenicity” in the cell death concept has contributed to this change. Recently, the Nomenclature Committee on Cell Death has described ICD as “a form of RCD that is sufficient to activate an adaptive immune response in immunocompetent syngeneic hosts”. In their report, the committee also pointed a number limited of agents that can promote this response [13].

Under normal conditions, an infection by a pathogen results in a programmed cell death, as a defense mechanism, named apoptosis. It has been shown that cells that undergo this type of cell death, largely mediated by the activation of caspases, are rapidly phagocytosed by APCs without eliciting an immune response. This type of cell death is considered tolerogenic because it lacks immunogenicity. On the other hand, necrosis was considered immunogenic due to the release of intracellular contents, provoking a pro-inflammatory milieu in absence of caspase activation [14]. Apart from these, autophagy, necroptosis, or ferroptosis, has been established as cell death mechanisms with overlapping processes and immunological significances [13]. Recent approaches have also changed the paradigm of apoptosis, relating apoptotic tumoral cells to the immune response. The use of some therapeutic agents and the study of the immunogenic consequences has opened new perspectives as “immunogenic apoptosis”. Based on a specific characteristic, different types of ICDs have been described:

- Pathogen-induced ICD: involves defense against bacteria and viruses. Upon infection by pathogens, cells detect PAMPs through specific pattern recognition receptors (PRRs). With this interaction, a warning signal is triggered that promotes autophagy with the consequent release of cytokines such as TNFs and type I IFN (IFN1). These pro-inflammatory cytokines activate APCs and the phagocytized infected dead cells are processed by them and are presented on MHC molecules. The presentation of these antigens by macrophages or DCs activates T cells (CD8+ and CD4+) for a long term adaptive immune response [15].
- Necroptotic ICD: This is a form of programmed cell death (different from necrosis that is consider “accidentally”) produce an irreversible plasma membrane permeation initiated by phosphorylation catalyzed by the serine/threonine kinase 3 (RIPK3) protein, which activates the pseudokinase mixed lineage kinase domain-like (MLKL) receptor, promoting the membrane dissolution [16]. Necroptosis is highly pro-inflammatory and is also capable of activating the adaptive immune system, generating a specific antigen response [15].
- Onco-therapy-induced ICD: This ICD is based on the exposure to anti-tumor agents and the activation of eukaryotic translation initiation factor 2A (eIF2A) phosphorylation of endoplasmic reticulum (ER) chaperones with subsequent membrane translocation of these intracellular proteins. Physical signals are also used to activate cellular immune responses. DCs exposed to dying cells due to chemotherapeutics, radiation or photodynamic exposure induce positive regulation of co-stimulatory molecules, release of pro-inflammatory cytokines and the closing of the immune cycle with tumor-specific CD8+ T cells recruitment [17].

**Figure 3:**
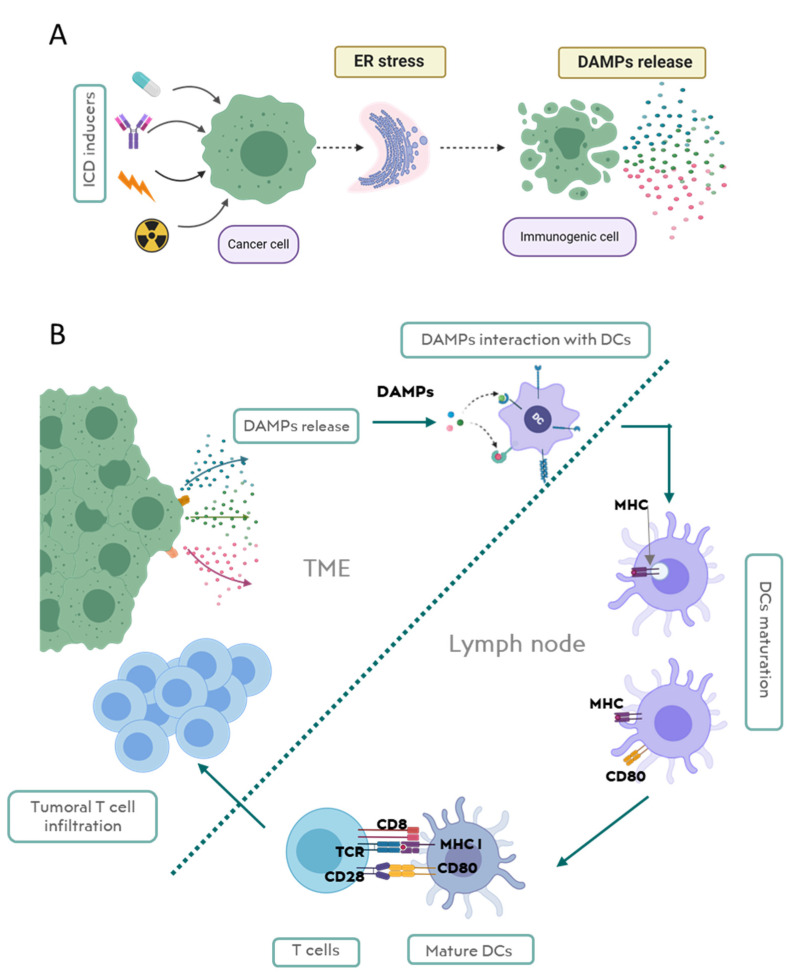
Major hallmarks in immunogenic cell death (ICD). (**A**) A Schematic representation of endoplasmic reticulum (ER) stress inducing damage-associated molecular pattern (DAMP) release. (**B**) Activation of immune machinery after DAMP release in ICD that culminates in the activation of adaptative immune response.

Regarding intrinsic ICD development, only some stimuli provoke a release of DAMPs by dying cells, which act as danger signals to produce immunostimulatory effects. Although this effect has been evidenced, it is still difficult to define which agents are responsible for the transformation from immune silent death to immunogenic death [18]. The common process that elicits DAMP release and the immunogenic response seems to be the ER stress [19]. The ER is a crucial organelle in homeostasis that is involved in folding and assembling of proteins. (Figure 3).

Cellular stress affects the ER directly with critical consequences in biochemical cellular machinery. As the ER’s role is pivotal for cell survival, a mechanism has evolved to detect and respond to stress, named the unfolded protein response (UPR). This mechanism restores protein folding capacity of ER by promoting chaperon expression and decreasing global protein activity. ICD is correlated with severe ER stress that is associated with inflammatory signals released to TME, alerting the immune system of cellular damage [20,21] (Figure 3A).

Therefore, DAMP release (or exposure) constitutes the danger signal alerting the immune system of this ER-associated cellular damage. DAMPs interact with APCs such as DCs promoting their maturation, migration, and interaction with T cells and activating adaptive immune system (Figure 3B). Here, we highlight the most representative DAMPs and discuss molecular mechanisms for the DAMP-mediated activation of the immune system in TME.

### 3.1. Calreticulin

Calreticulin (CRT) is a soluble protein related to the homeostatic Ca^2+^ control of the cell. This biomolecule is a highly conserved protein of 46 kDa that was firstly associated with the ER where it plays a role as a chaperone, involved in the regulation of protein synthesis [19]. In the ER, it interacts with ERp57 and calnexin to help in the proper folding of proteins. Additionally, CRT seems to be crucial in the assembly of MHC class I and the antigen presentation before cell surface presentation [20]. Furthermore, CRT appears in the nuclear envelop as a transporter protein while its presence in the cytoplasm has not yet have a biological role defined [21,22].

The significance of CRT in ICD is its translocation and the exposure on the cell membrane (ecto-CRT) making CRT as one of the main representative DAMPs [19]. In spite of early indications about its immune-related role, the induction of immunogenic apoptosis by ecto-CRT exposure must be linked to further apoptotic events because the presence of ecto-CRT alone is not sufficient to activate the immune response [23].

**Figure 4:**
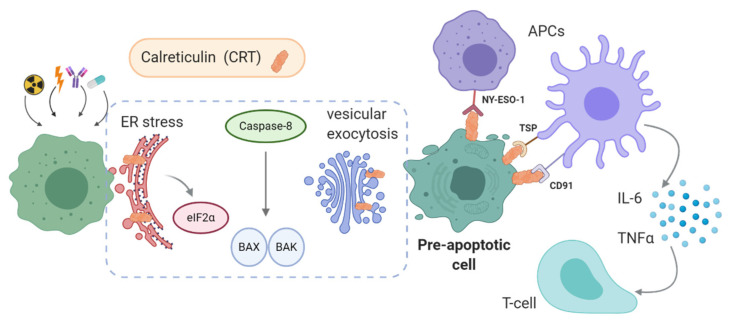
Schematic representation of calreticulin (CRT) exposure and its interaction with the corresponding receptor in ICD response.

The increase of the intracellular ROS concentration mediated by anti-tumoral drugs leads to ER stress caused by misfolded proteins, and induces the exposure of CRT (Figure 4) [24]. The pERK-dependent downstream signaling is activated by ER-stress, resulting in eIF2α phosphorylation. The apoptotic pathway is mediated by caspase-8 following the activation of pro-apoptotic proteins BAX and BAK. This pathway promotes the mitochondrial external membrane permeabilization and the transport of CRT from the ER to the Golgi apparatus, where CRT is exposed through vesicular exocytosis [25].

These events are demonstrated to be essential for ecto-CRT-mediated ICD. Experimentally, this sequence of events was confirmed by the administration of antioxidants or with the knockdown of proteins participating in apoptosis such as PERK or caspase-8 [26]. Also, antibodies blocking ecto-CRT, gene knockouts of the protein, and inhibitors of phosphorylation of eIF2α eliminate the ICD observed after tumor treatments [19,27]. Some authors also point out that there is a relationship between the production of cytokines such as IL-8 by the cells and the exposure of CRT [28].

Ecto-CRT has emerged as a pre-apoptotic signal similar to the phosphatidylserine (PS) exposure but with several differences [29]. Ecto-CRT appears before PS in apoptotic cascade and acts as an “eat me” signal for APCs such as DCs, triggering an immune response. In the case of PS, it mediates the elimination of apoptotic cells in an immunologically silent manner [19].

In fact, ecto-CRT acts as a pro-phagocytic signal promoting the engulfment of cancer cells by DCs and the release of IL-6 and TNFα [30]. Other interactions of CRT also promote the phagocytic functions. Thrombospondin (TSP) or CD91 interaction on the APCs, stimulates the engulfment of apoptotic cells. Additionally, CRT interacts with NY-ESO-1 on the cell surfaces of DCs and macrophages. This receptor, used for developing new immune-based therapies, is involved in the adaptive immune response against tumor cells with CD8+ T lymphocytes [31].

A representative drug class that induces ecto-CRT are anthracyclines. In fact, these compounds have served as a prototype to elicit the molecular mechanisms underlying the ICD. Within the anthracycline family, many natural products are included. Considering its coplanar structure, these molecules act as DNA intercalants as well as topoisomerase II (Topo-II) inhibitors. Furthermore, the presence of a quinone structure makes these compounds capable of triggering oxidative stress in cells [32]. It has also been observed that platin derivatives also induced ecto-CRT in vivo and in preclinical studies in different tumors [33,34]. Other therapeutic strategies applied in cancer such as radiotherapy use this mechanism and will be widely covered in this work [35].

### 3.2. HMGB1

**Figure 5:**
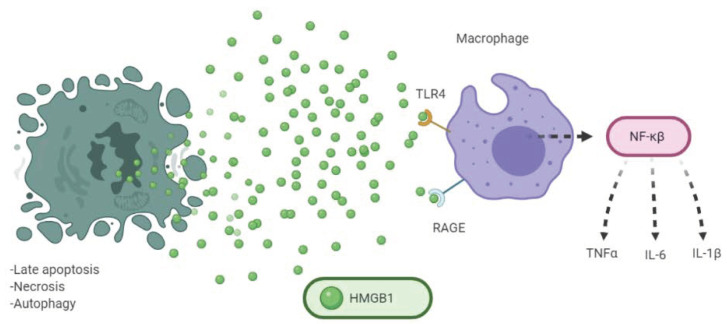
Implication of HMGB1 in cell death and activation of adaptative immune response.

The high mobility group box 1 protein (HMGB1) is a nuclear protein that physiologically acts as a chromatin-binding protein. In the nucleus, HMGB1 interacts with DNA assisting in the formation of protein complexes and carries out different functions like transcription regulation of p53 or NF-κβ [12]. Structurally, it is a single polypeptide chain with two N-terminal globular domains that bind to the DNA, HMG boxes A and B, where the B box is important for the immune properties of the protein [36]. Unlike the other proteins of the family with which it has a high percentage of structural similarity, HMGB2, and HMGB3, this protein is expressed ubiquitously and beyond the embryonic period [20]. It is worth mentioning that post-translational modifications (PTM) or oxidative status of HMGB1 seem to be particularly important for its interaction with receptors [37]. Apart from the role played in the nucleus, it can be secreted by activated macrophages and monocytes after the release of inflammatory factors such as TNFα, LPS, or IL-1β. A lysine acetylation is required that confers the chemical characteristics to be passively secreted from the nucleus to the extracellular medium [38]. HMGB1 mediates ICD as a DAMP when released from dying tumoral cells in the late stages of apoptosis, necrosis, and autophagy. The first characterization of HMGB1 was in the necrosis process, where the concentration of the extracellular protein is significantly abundant. Comparatively the amount of HMGB1 secreted in apoptosis is lower than it is in necrosis, however, the response caused by macrophages in the absorption of dead tumor cells, again induces an active release of HMGB1, with the consequent accumulation of this protein in TME. Immune activation mediated by HMGB1 is due to the interaction with different PRRs such as toll-like receptor (TLR) 4 and receptor for advanced glycation end products (RAGE). TLR family is responsible for innate immune response activating inflammatory reactions through the release of TNF-α, IL-1β, and IL-6. TLR4 is a prototypical member of these proteins and the interaction of HMGB1 with this receptor elicits the stimulation of NF-κβ and the production of cytokines IL-6 and TNF-α by macrophages [39]. On the other hand, RAGE is an immunoglobulin protein with a transmembrane structure that recognizes HMGB1 (mainly their disulfide form) and promotes the activation of the NF-κβ pathway as well as the MAP/ERK kinase cascade (Figure 5) [40].

As mentioned above, the oxidation status of the biomolecule affects the interaction with receptors, in particular, a sulfur reduced form in residue Cys-106 is necessary for proper coupling with TLR4 [41]. This fact constitutes negative feedback for HMGB1 as the release of pro-inflammatory cytokines in the presence of the protein promotes oxidative TME conditions [42]. Regarding the sulfur bonds, a disulfide bond between Cys-23 and Cys-45 increases the biological stability of the protein, and the substitution of these positions for alanine or serine residues decreases the cytokine-like properties of HMBG1 [43]. Hence, this evidence demonstrates a PTM-related oxidation of HMGB1 that autoregulates the pro-inflammatory properties in TME and should be considered in experimental protocols or clinical practice.

### 3.3. ATP

The intracellular role of ATP as the main metabolic energy source is well-known, however, the functions of extracellular ATP have also been described from early on with vasoactive and antiplatelet effects [44]. In the context of ICD, ATP in TME works as a “find me signal” for chemotaxis of myeloid-derived cells [45]. Interaction of ATP with purinergic receptors P2 is responsible for the immune adaptative response mediated by the nucleotide. This family of receptors is divided into subfamilies: P2YRs, G-protein coupled receptors with a metabotropic activity, and P2XRs which are ligand-gated ion channels. P2RY2, one of the receptors that belong to the former class, is found in APCs like macrophages and it promotes their maturation and the release of cytokines. As a consequence of this interaction, T cells infiltrate the tumors. Similarly, ATP acts as a molecular signal to open ion channels of P2X7R on APCs releasing pro-inflammatory factors and gathering immune cells in TME [46]. This last immunostimulatory function of ATP is due to the activation of NLRP3 inflammasome that is characterized by the release of IL-1β and the consequent priming of CD8+ T cells [26]. Finally, ATP exerts an immune response in the TME by also increasing NK cell activity [12].

ICD triggered by ATP mainly depends on the concentration. ATP in the extracellular milieu should reach mM range to switch on the enginery of T cells while long exposures of small amounts of ATP in the TME can cause incorrect maturation of DCs [47]. ATP signaling expires in a short time (seconds to minutes) by the enzymatic transformation of the nucleotide to ADP and AMP through the ectonucleotidases CD39 and CD73 expressed on Treg cells. As stated before, ATP activates the immune system through several pathways, however, it is worth mentioning that ADP possesses immunosuppressive properties and can be a promoter of tumor progression in TME [48].

**Figure 6:**
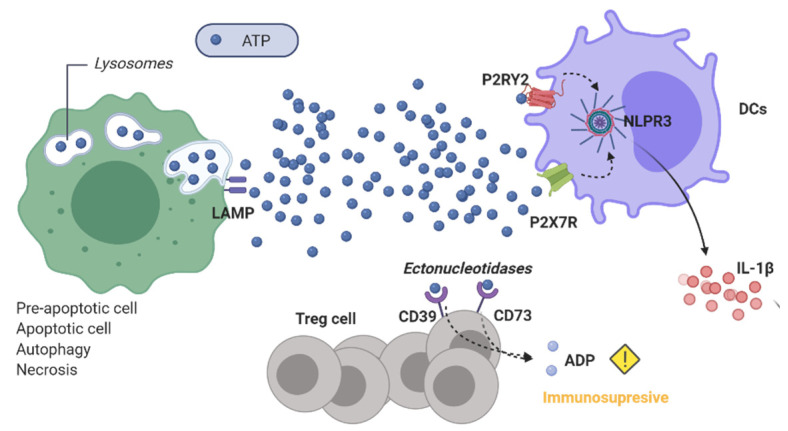
Release and the role played by ATP as a DAMP in TME.

Regarding the molecular cell death pattern, ATP release can be triggered by different intracellular pathways in ICD. In the same manner as HMGB1, ATP release occurs passively when the cells reach the necrotic state and there is an increase in permeability of cellular membranes that provide the diffusion of ATP to the TME. The release of ATP was also described in apoptotic progress. As CRT, ATP can appear in pre-apoptotic stages when anti-tumoral treatments induce ER stress that activates PERK signaling and the PI3K-dependent trafficking of vesicles to the extracellular milieu [49]. ATP can also be secreted in the early stages of apoptosis during the cell disassembly mediated by PANX1 [50]. The mechanisms involved in ATP release during apoptosis are closely related to lysosome metabolism. This nucleotide is stored in the cell in this organelle and for this reason, the release of ATP is closely related to the autophagy process. ICD-related release of ATP requires not only apoptosis cascade mediated by caspase activation but also the translocation of lysosomal-associated membrane protein (LAMP) 1 to the cell surface (Figure 6) [51].

### 3.4. Other DAMPs

**Figure 7:**
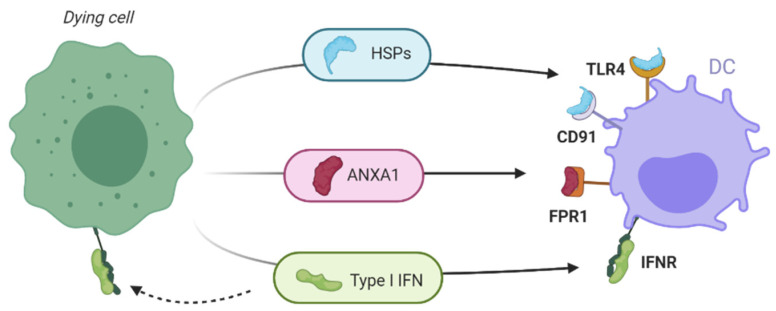
Other DAMPs involved in ICD.

Dying cells release other biomolecules that can mediate a pro-inflammatory response when interacting with DCs (Figure 7). Heat shock proteins (HSPs) are chaperons appearing in cells in response to a physical stimulus (increase of temperature, osmotic pressure or pH change) as protective proteins in charge of repairing or eliminating other proteins affected by the stress produced by the stimulus. Therefore, their presence in the cytosol could be considered an anti-apoptotic mechanism [52]. As it was described previously in this work for CRT, the release or exposure of HSPs increases tumor immunogenicity [53]. Accordingly, exposure of HSPs such as HSP70 (heat shock 70 kDa protein) or HSP90 (90 kDa protein) mediate the maturation of DCs through different interactions [54]. In APCs, HSPs bind to CD91 and promote the engulfment of apoptotic cells and hence, increase the uptake of dying cells and prime the CTL by interacting with TLR4 on APCs [19]. As soluble proteins in TME, they possess a cytokine-like function boosting effect on NK cells and stimulate NF-κβ with the corresponding release of soluble effectors such as IL-6 and TNF-α [55].

Homeostatic regulatory functions have been assigned to annexin A1 (ANXA1) but it has also been demonstrated that this protein can acts as a DAMP by inducing inflammation when it is passively released to the TME by necrotic cells. The interaction of ANXA1 with the formyl peptide receptor 1 (FPR1) in immature DCs promote the uptake of tumor antigens. Lack of functional ANXA1 or single polymorphism in FPR1 in patients hinders the immune response mediated by ICD inducers [56].

IFN1 is a family of polypeptides that acts as innate immunomodulatory agents against microbial infections. IFN-α and IFN-β are the prototypical members of this family and they have a role in innate and adaptive immunity [57]. Cytolytic responses mediated by activation of CD8+ T cell and NK is enhanced by the engagement of DCs through TLR receptors. On the other hand, IFN1s inhibit the immunosuppressive function of Treg cells promoting immune surveillance in TME. Expression of membrane IFN receptors (IFNR) by cancer cells makes them susceptible to binding by IFN1s, so that, there can be a continuous feedback in TME that enhances the ICD. This also promotes the response mediated by cytokines. IFN1 also mediate release of C-X-C motif chemokine ligand 10 (CXCL10), a powerful chemotactic factor [58].

## 4. Targeting TME: ICD Inducers

ICD inducers are a heterogeneous group including a large number of different organic compounds, biological therapies, and physical methods for the treatment of cancer. Therefore, it is difficult to make a classification of ICD inducers by structural relationship, source, or target. As stated in the section before, one factor in common for ICD is the in-duction of ER stress. As this phenomenon seems to be the intracellular starting point for DAMPs release and the consequent immunostimulation, the capability of treatments to provoke ER stress has been used to classify the ICD inducers [12]. Most recognized ICD inducers (Table 1) have specific targets in the cytoplasm, nucleus, or membrane, and trigger ER stress as a consequence of their interaction with their pharmacological target. These agents can be classified as type I ICD inducers and their cell death pathway is not mediated by ER but ER stress appears once they activate their pathway signaling. On the other hand, type II ICD inducers may focus their pharmacodynamic properties in producing ROS for ER stress directly (Table 1) [12]. The ability to produce the specific ROS-ER tandem as a pharmacological response seems to elicit a more effective and faster ICD with a high number of DAMPs detected in TME. Both types of inducers can have in common production of ROS since the immunogenicity of cells was decreased in the presence of antioxidant agents [24]. However, Type I ICD inducers with this capacity require more studies to determine the relationship between these species and ER stress. More recently, Aaes and colleagues reported an immunogenic necroptosis triggered by Type I inducers not mediated by ER stress that contribute to the idea of an alternative mechanism for these agents to elicit ICD [59].

In parallel with these concepts, in literature the idea of bona fide inducers also appears, referring to those therapies inducing an efficient response in immunocompetent individuals but fail when they are tested in immunocompromised murine models. Moreover, bona fide ICD inducers should be able to produce “vaccines” when cancer cells are treated with these agents and enhance immunogenic responses only in immunocompetent hosts [60].

Both concepts mentioned above highlight the difficulty in characterizing ICD inducers. This fact, together with the relatively short trajectory of immunogenic death research, makes it difficult to establish standards for the administration of this type of therapeutic agent. However, in recent years much progress has been made in sharing the results of therapies that induce a response because of tumoral cell death. Here, we have collected different chemotherapies as well as other types of anti-tumoral therapies that are growing in importance as ICD inducers thanks to their influence on TME immune conditions. In this work, several chemotherapeutics have been reviewed and classified according to their origins: chemotherapy, monoclonal antibodies, oncolytic viruses, and physical modalities for cancer treatment

### 4.1. Chemotherapy

**Figure 8:**
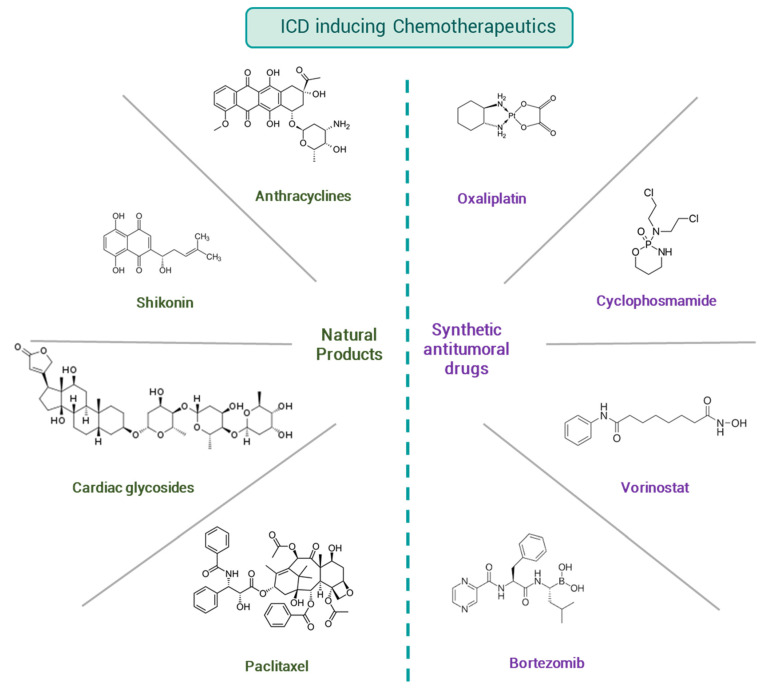
Drugs from natural sources and synthetic approaches that induce ICD.

The term chemotherapy refers to the administration of cytostatic or cytotoxic drugs with the aim of disrupting the unlimited proliferation of cancer cells. Chemotherapeutics target both membrane receptors and a multitude of intracellular biomolecules, among others. Herein, we discuss drugs from natural sources and synthetic ones that have their anti-tumoral mechanism of action, while also inducing ICD (Figure 8).

#### 4.1.1. Natural Products

Natural sources have played an essential role in drug discovery. Particularly in chemotherapy, more than 60% of approved drugs are obtained from natural products or their rational design. Structural variability involving natural products enable them to have heterogeneous pharmacological activities [61]. Therefore, it is not surprising that among ICD inducers, numerous natural products appear as discussed in this work.

##### Anthracyclines

These drugs are an important class of antibiotics obtained from *Streptomyces* sp. and used in the treatment of numerous types of cancers since the first administration as anti-tumor agents in the 1960s. From the discovery and characterization of the first anthracycline, Daunorubicin, a complete structure-relationship has been built, giving rise to the most representative chemotherapeutic agent from the family, Doxorubicin [62]. Several pharmacological mechanisms are involved in the cytotoxicity of anthracyclines. The coplanar rings allow anthracyclines to intercalate between the base pairs of DNA, decreasing cellular replication and inhibiting Topoisomerase II. Also, the quinone structure in the skeleton leads to the formation of ROS that is correlated with the modulation of transcription factors that control proliferation.

Anthracycline and its analogs were one of the first agents to be known to have an immunogenic response after treatment, and later on, many studies reported their effect on ICD. First studies in determining ICD mediated by doxorubicin was carried out by Kroemer’s group where the apoptotic response of this drug was employed to develop a cancer vaccine based on the proadministration of doxorubicin and the subsequent activation of the immune system against the tumor [63]. ICD mediated by anthracyclines was also described together with their ability to expose CTR on the cellular membrane by Obeid and collaborators. They described the translocation of CRT in a murine model when cells were treated with doxorubicin and the subsequent engulfment by DCs, leading to TAAs presentation and tumor-specific cytotoxic T lymphocyte responses [19]. The release of HMGB1 and HSPs mediated by anthracyclines has been also characterized in a work on different solid and hematological cancer types [64]. ATP release mediated by anthracyclines was determined by several reports where they confirm the binding of the nucleotide with the purinergic receptors in DCs that promote their activation. Tumor-infiltrating leukocytes activated in an ATP-dependent manner were also described [65,66].

Humoral response is also identified after treatment with this type of drug. In another report, the production of IL-17 was shown when cells from different cancer types (i.e., colon, sarcoma, and breast) were exposed to mitoxantrone, an anthracenedione closely related to doxorubicin [67]. In cellular response, anthracyclines play an important role as they modulate T cell activation and tumor-infiltration as was reported in several murine models of solid tumors [68]. Another recent report has continued the search for new molecular mechanisms that explain the relationship between ICD and anthracyclines. In this work, it is outlined how the systemic induction of autophagy, a cell death mechanism linked to ICD, improves tumor regression with this pharmacological treatment by reducing the toxicity associated with these drugs [69]. The efficacy of this type of treatment in combination with other therapeutic agents for their ICD-inducing action is evidenced by the numerous active clinical trials involving doxorubicin and other anthracyclines such as epirubicin. In a recent publication by Vanmeerbeek, it can be observed how these drugs are included as components in standard regimens with other chemotherapy agents or with immunotherapy [70].

##### Shikonin

Shikonin is a naphthoquinone isolated from the roots of *Lithospermum erythrorhizon.* Extracts from this plant have been widely used as a topical formulation for several dermatological diseases in traditional medicine. Once the pure enantiomeric form was isolated in the 1980s, the inflammatory and cytotoxic properties of these molecules have been studied [71]. Apoptosis induced by this natural product is mediated by ROS generation [72], which can be correlated with its capacity to induce ICD. The presence of the quinone group in the structure, as occurs in anthracyclines and their analogs, can be the reason for this activity. Moreover, shikonin also shares with some anthracyclines other molecular effects such as Topo-II inhibition [71]. The immunogenic activity was reported by Chen and colleagues in a work where shikonin was compared with doxorubicin and other ICD inducers in a melanoma tumor model in mice. In this approach, authors also characterized apoptosis as well as humoral (ILs and IFNs) and cellular response triggered by the drug [73]. Other authors also characterized necroptosis as immunogenic cell death mechanism of shikonin [74]. A cancer vaccine based on the administration of shikonin has shown encouraging results: treatment of murine breast cancer cells enhanced the antimetastatic effect as compared to doxorubicin used as chemotherapy control. [75]. Recent studies with integrated transcriptomic and metabolomic data of an animal model treated with shikonin confirmed the ROS pathway activation and the correlation with its necroptotic effect, providing new perspectives into exploring the anti-cancer potential of this drug as ICD inducer [76].

##### Cardiac Glycosides (CG)

Digoxin and digitoxin are two drugs extracted from *Digitalis lanata* and *D. purpurea* that are generally used as anti-congestive and antiarrhythmics in heart failures. These compounds promote the increase of intracellular concentration of Na^+^ and Ca^2+^ and the decrease of K^+^ by inhibiting the membrane Na^+^/K^+^-ATPase pump. Anti-tumoral properties of CG have been described for a long time [77] and has been widely reviewed [78,79]. More recently, their effect as ICD inducers have been included among their potential action in chemotherapy based on the alteration of Ca^2+^ homeostasis. Accordingly, it was shown that CGs enhanced anti-tumoral effects of chemotherapeutic agents that do not induce ICD, only in immunocompetent mice [80]. In fact, their anti-tumoral effect has been linked to DAMP release that activates the immune response [81] and improved the immuno-genic profile of drugs lacking this property such as cisplatin or mitomycin C [82]. Other cardiac glycosides structurally related with digoxin, bufadienolide derivatives, have been found to be selectively cytotoxic against certain glioblastoma and pancreatic tumor cell lines. The administration of low doses of these natural compounds not only produces tumoral cell death but also provokes the depletion in Treg cell population. This fact could propose these compounds as candidates for adjuvants in anti-tumor treatment by promoting changes in the cellular response of the TME [83]. The widespread and safe use of drugs such as digoxin has made their clinical application possible as potential ICD inducers [84].

##### Paclitaxel

Paclitaxel (PTX) was discovered in *Taxus brevifolia* in the 1970s and nowadays, it is a drug included in clinical anti-tumoral protocols of different types of cancer. Molecularly, PTX targets tubulin of microtubules, stabilizing mitotic spindle and preventing disassembly. It has been suggested that the polyploidization derived from this effect may be the cause of ER stress triggered by PTX as it promotes CRT exposure. The apoptosis mediated by the antimitotic effect promotes an immunogenic response [85]. Other work also showed the relationship between the response to PTX and the release of other DAMPs such as HMGB1 [86]. The effect of combining this antimitotic as an adjuvant with an oncolytic virus was tested and how PTX promoted the cytotoxic effect of the virus was investigated. Low doses of this antimitotic with the virus induce aberrant mitosis that synergized with the virus activity in xenografted ovarian cancer in mice [86]. Maturation of DCs and TLR4 activity was tested in mice treated with PTX and cyclophosphamide. This report also shows a connection between a low dose of PTX and T cell recruitment and activation together with cytokine release in mice [87]. This study was corroborated by the report of Lau and collaborators that detailed the implication of TLR4 receptor in ICD induction by PTX with an entire description of the signaling pathways associated [88]. This work also showed DAMP release mediated by this antimitotic drug promoted ICD. Finally, authors described a cellular immune response mediated by the injection of cells pretreated with PTX in a murine model of ovarian cells [88]. This description highlights the importance of PTX in ICD and promotes the search for new mechanisms involved and possible new applications of this drug that is widely used in conventional chemotherapy.

#### 4.1.2. Synthetic Anti-Tumoral Drugs

Among the wide variety of treatments available to target tumor cells, there are approved synthetic drugs with demonstrated ability to induce ICD. Once again, they constitute a very diverse group in which it is difficult to establish a relationship between their immunogenic activity, their mechanism of action and their chemical structure.

##### Oxaliplatin

Oxaliplatin (OX) is a third-generation platinum-based agent that forms platinum-DNA adducts and induces apoptosis. OX is approved in clinic for the treatment of metastatic colon carcinoma, where its analog cisplatin is ineffective. This approval has substantially improved the quality of life of patients whose treatment regimens combine OX with drugs such as 5-fluorouracil [89]. From early on, there are indications that OX is an inducer of ICD. Some of the DAMPs and receptors associated with ICD have been described and characterized and confirmed as hallmarks of ICD using this drug as a reference. This is the case for CRT exposure, the release of HMGB1 and ATP and the role of TLR receptors [23,90,91]. Furthermore, the study by Sato and collaborators shows how the use of OX in patients reduced the population of Treg cells and enhanced CD8+ T cells in ovarian cancer [92]. The same results were reported in a colon carcinoma mouse model where levels of macrophages and myeloid-derived suppressor cells (MDSCs) were also explored, concluding that administration of OX led to a less tumor immunosuppressive microenvironment by the reduction of Treg cells [93]. An interesting data about the dose of OX to serve as ICD inducer was reported by Roberts and their group in glioma cells. Low doses of OX showed remarkable results on intracellular pathways like eIF2α phosphorylation, in macrophage-mediated response and the inhibition of pro-tumor factors in TME [94]. The recent studies carried out in comparison with cisplatin also shed light on the ICD induction by OX. Some previous reports had not attributed changes in tumor immunogenicity by the lead compound [19], however, the work carried out on head and neck tumors showed the comparison of both drugs were able to promote CRT and HSP70 exposure [95]. This trend regarding the use of low doses of OX has also been reflected in other work where nanotechnology has been used precisely to promote a controlled and constant release of OX doses [96]. Recent advances in the use of OX as an ICD inducer point to a potential combination of this drug with immunotherapy in murine models. A study carried out in different colon carcinoma animal models revealed that the pre-treatment of the mice with OX prevented resistance to the immunotherapies for certain murine colon carcinoma cell lines [97]. In the same line of research in lung cancer, it has also been possible to observe an increase in the activity of PD-L1 inhibitors with pretreatment with OX by the promoting of an immunogenic TME mediated by the platinum-derived drug [98].

##### Cyclophosphamide

Cyclophosphamide (CPA) is a nitrogen mustard alkylating agent, often used in combination with other therapeutics, for the treatment of numerous solid tumors such as ovarian cancer, breast cancer, small cell lung cancer, and sarcoma; and in hematological diseases including Hodgkin and non-Hodgkin lymphomas, myelomas and leukemia [99]. Closely related in structure and metabolism with CPA is the drug mafosfamide (MAFO). Both, CPA and MAFO, are highly metabolized by cytochrome P450 into the same derivates: phosphoramide mustard and acrolein. In concordance, when the immunogenic reaction mediated by this drug was characterized, it was possible to observe the exposure of CRT in the membrane at the beginning of cell death as well as the liberation of HMGB1 in the final phases of the process, in the same way as the leading compound CPA [100]. In terms of immunomodulation, high doses used in the clinical protocols of CPA cause a strong lymphodepletion [12]. First reports that correlate CPA with immune activation in TME have found evidence of the decrease in the levels of immunosuppressive levels of cytokines IL-10 and TGF-β together with the increase in CTL activity [101,102]. Later, studies have shown that their best immunomodulator effects arise when administered in low doses, as in the case of OX. In cellular immune activation there are some examples of the employment of metronomic doses to trigger ICD response. In a study of brain xenograft tumors treated with low doses of CPA, several biomarkers that are related to cellular immune responses were identified, such as CD68 and CD74 attributed to macrophage and DCs activation, respectively. In humoral response, several types of IFN mediators and cytokines were also identified after treatment with CPA [103]. They also detected the increase of factors related to HMGB1 and its related receptor such as RAGE. They conclude their study remarking the importance of their results to explain the mechanism of metronomic doses of CPA but also highlighting the importance of the findings as prognostic biomarkers in this type of treatment [103]. One interesting effect of low doses of CPA is the depletion of levels of Treg cells. This result, like the report of Ghiringhelli and collaborators and from Audia and collaborators, demonstrate this effect in patients with advanced stages of the disease [104,105]. In summary, molecularly and experimentally, CPA has a good profile for being applied as an adjuvant in other chemotherapies due to the clinical status of the drug, although it seems essential to consider the dispensed dose if a modulation of the immune response in the TME is sought.

##### Bortezomib

Approval of bortezomib was a breakthrough in the treatment of multiple myeloma and mantle cell lymphoma. This was the first ubiquitin-proteasome inhibitor introduced in clinical practice that changed the paradigm of therapeutic targets by blocking a biomolecule involved in regulating protein stability and thus normal cellular function [106]. Bortezomib is a reversible inhibitor of the 26S subunit of proteasome, promoting the protein homeostasis disruption, ER stress, and consequently, apoptosis [107]. In a myeloma model, the immune DC stimulation was described by Spisek and collaborators, where they were able to verify the activation of enhanced autologous anti-tumor T-cell response to primary human tumor cells after apoptosis mediated by bortezomib [108]. These authors also correlated the immune response with the appearance of HSP90, as well as CRT, in the cell membrane, when these phenomena had not yet been proposed as ICD key points [108]. Cellular immune activation was also characterized in response to bortezomib in a later report. Base on a murine ovarian tumor model, the authors described tumor regression after injection of cells loaded with bortezomib. In this work a complete description of T cell tumor-infiltration and DCs maturation progress was described. Authors also demonstrated the importance of protein HSP60 in the immunogenic process, which completes the set of DAMPs that can be observed by the action of bortezomib [109]. Other reports focused on equilibrium of immune soluble factors where bortezomib tends to increase inflammatory cytokines while decreasing the antagonist of this anti-tumor reaction [110]. They also described the activation of intracellular pathways that together with cytokine stimulation, promotes CD8+ T cells activation after administration of bortezomib [110]. This preclinical evidence together with the drug’s track record in clinical practice may promote the use of this drug as an adjuvant to enhance the anti-tumor immune response. Recently, clinical trials of bortezomib in combination with other therapeutic agents have been initiated that could reveal more data about the applicability of the immunogenicity of this drug [70].

##### Histone Deacetylase Inhibitors

The understanding of epigenetic modifications and their importance in the development of tumors has made it possible for histones to become a new target in drug design. The use of histone deacetylase inhibitor (HDACi) in preclinical trials has given good results, however, the application in clinical has not been as satisfactory [111]. Nevertheless, in spite of the obstacles in the development of these drugs, vorinostat, a HDACi was introduced in the clinic for the treatment of refractory cutaneous T-cell lymphoma [112]. HDACis have shown an increase of anti-tumoral activity of other drugs when they are used in combined administration guidelines [111]. Immunogenic characteristics has been attributed to HDACi and were widely reviewed by Shakespear and collaborators [113]. This property is also attributed to vorinostat and can be correlated with ICD. One relevant characterization of ICD hallmarks was conducted by Sonneman and colleagues. This group described the exposure of CRT by vorinostat treatment in different childhood tumor cell lines of brain and sarcoma pathologies [114]. They concluded that this drug can translocate CRT to the cell membrane as other ICD inducers, depending on caspase-apoptotic pattern triggered during cell death process [114]. Later, the work from West and collaborators reveled the release of HMBG1 and ATP in apoptotic response triggered by vorinostat. Cellular components were also characterized in this study carried out in a murine model of colon adenocarcinoma. Authors remarked on the importance of B lymphocytes in the immune response observed with this HDACi as the B cell-deficient mice had a shorter survival upon vorinostat treatment as compared to the wild type mice [115]. The most recent reports point to the use of vorinostat in combination with other current immunotherapies. A combination with the anti-PD-1 pembrolizumab is in clinical trials (phase 1) in patients with advanced non-small lung cancer. These patients presented tumor-infiltrating lymphocytes that were correlated with the administration of the drug [116].

### 4.2. Monoclonal Antibodies

Administration of monoclonal antibodies (mAbs) for targeting cancer is a widespread strategy today. The clinical use of trastuzumab or cetuximab against epidermal growth factor receptors (EGFRs) has opened new drug development opportunities as well as improved patient prognosis. By blocking this family of tyrosine kinase receptors that are overexpressed in different types of tumors, the downstream signaling involved in uncontrolled cell proliferation is also inhibited. It has also been shown that there are immune responses associated with this pharmacological activity [117]. In the search for new therapeutic entities with the same mechanism of action, 7A7 emerged as a therapeutic analogue of cetuximab in mice. Garrido and collaborators in the early 2000s described the specifity of this mAb in murine models and proposed its potential for preclinical trials [118]. In the first report, a high long-term apoptotic response was attributed to 7A7, suggesting that it induces ICD. Accompanying the description of apoptosis mediated by this mAb, they also found increased CD8+ tumor infiltration signals and the exposure of CRT in the extracellular membrane [119]. A little later, the same authors proposed their own preclinical model for the study of 7A7. This model confirmed the previously described T cell activity and provided interesting insights into the expression of MHC-I molecules. In the animal model, there was either a complete loss of MHC-I molecules or a decrease in expression due to defects in IFNγ-mediated induction. In this case, exceptionally low levels of mRNA for MHC-I or loss of IFNRs expression were detected [120]. These results along with others reported by He and colleagues, cast doubt on the ICD induction of 7A7 [121].

Nevertheless, another tyrosine kinase inhibitor receptor was studied by Pozzi et al. In this case, the mAb cetuximab approved for different types of cancer with EGFR overexpression was studied with respect to the induction of ICD. A murine model of colon cancer was used as well as many cell lines related to this type of cancer which provide the authors with the interesting conclusion about the immunogenic response [122]. Thanks to their analysis, they could conclude that the presences of mutation in downstream signaling of RAS play an important role in ICD induced by cetuximab and explain the failure of this treatment in certain types of colon carcinomas [122]. Another work with patient samples of colorectal cancer have also demonstrated the immunogenic properties of cetuximab in TME as evidenced by enhanced immune cell infiltration into the tumors studied [123].

### 4.3. Oncolytic Viruses

Oncolytic virotherapy described by Kir in 2001 refers to selective virus replication within human tissues for anti-tumoral effect [124]. The initial clinical trials revealed the potential of these therapies in combination with chemotherapy. This concept has progressively evolved into the idea of oncolytic immunotherapy and has become an effective tool to modulate the immune response in TME [125]. Oncolytic viruses (OV) can be used to produce tumor antigens for T cell activation or to induce anti-tumoral immunity by infecting the tumor cells. ER stress was described for the coxsackievirus B3 infection as its main mechanism of action. These facts promoted the inclusion of this kind of therapy as one of the type-II ICD inducers [12]. Once these therapies were recognized for their immunogenic potential, the last few years have been very successful in the description of immunomodulation by OVs.

Some examples of different apoptosis mediated by OV revealed the correlation of the immune response with the exposure and release of DAMPs after infection of different cell lines. Infection of squamous carcinoma cells with herpes virus has shown that the immunogenic response observed corresponds with ICD thanks to the characterization of the CRT exposure and the release of HMGB1 and ATP [126]. The study of the JX-594 OV in melanoma cell lines carried out by Heinrich and collaborators also reveal the triggered ICD with the characterization of soluble DAMPs [127]. Later co-culture of infected melanoma cells with DCs and cytotoxic lymphocytes showed the maturation and activation of the former cell population but failed in the recruitment of the latter. However, the author could conclude that the proposed therapy improved the immunogenic properties in TME [127]. The study of coxsackievirus A21 also demonstrated the ICD induction. In this case, the study was centered on bladder cancer cell lines and a murine model derived from MB49 cell line transfected with the viral entry receptor. In this study, CRT exposure and HMGB1 release could be detected but not ATP release. An in vivo study was also carried out that has shown that cell lysates pre-treated with the OV acts as an effective vaccine that led to tumor rejection in syngeneic mice. Newcastle virus has also emerged as an OV with a good therapeutic profile and as an ICD inducer. A study on human lung cancer cell lines have shown that Newcastle virus induced ICD in an autophagy-dependent manner and in xenograft model of lung cancer mice treated with the supernatants of the virus infected cells could control the tumor growth [128]. Novel approaches of combining administration of OV with other anti-tumoral therapies to take advantages of its immunogenic properties in TME have been gaining popularity. The different OV options described so far have been thoroughly reviewed by Zhang and Cheng recently together with challenges in therapies, different administration with immunotherapy or cell therapy as well as novel strategies in virus engineering [129].

### 4.4. Physical-Chemical Methods for Cancer Therapies

#### 4.4.1. Radiotherapy

Radiotherapy (RT) is a cancer treatment based on high doses of ionizing radiation that targets DNA replication leading to mitotic catastrophe and, consequently, to cell death. Radiation plays a role not only in the direct treatment of tumors but also as a neo-adjuvant to reduce tumor size before surgery or after interventions to eliminate the remaining cancer cells [130]. Proliferating cells such as tumor cells are more sensitive to RT effects as their replication machinery is enhanced, however, localized treatments are the current approach to avoid side effects. In this context, it is worth mentioning the “abscopal effect” mediated by this therapy, which involves regression of tumors in distant parts from the place targeted by radiotherapy. This effect represents the main evidence for ICD induced by RT that can be exploited in TME modulation [131].

As an immunogenicity inducer, RT elicits a cellular and chemotactic response in TME where it promotes the release of cytokines and chemokines and recruits immune cells. Firstly, dose-dependent RT response for IL-1 induction was observed in a murine model [132]. In terms of MHC-I expression, the enhancement of the protein pool mediated by RT seems to be the primary reason for the increase in the number of these molecules. The same authors also reported that there is a late effect mediated by the mTOR pathway activation that also enhances the MHC-I presentation [133]. Furthermore, RT was reported to be involved in the release of CXCL16 that activates T cells in different types of cancer [134].

Today, it is well-known that RT and DAMPs are closely related and the exposure/release of the main hallmarks of ICD is perfectly characterized. CRT exposure was detected in vitro and in vivo models of prostate cancer when treated with RT, while CTLs were also more sensitized to killing tumor cells. The response was also linked with the other DAMPs, HMGB1 and ATP, which are inherent to the RT immunostimulation [135]. Intracellular HMGB1 and released HMGB1 levels are correlated well with RT treatment. Autophagy induced by HMGB1 is a strategy to avoid RT resistance, so that, this DAMP became a biomarker for a correct exposure regimen in RT. However, the dose of the ionizing agent should be properly adjusted to avoid the change in the immune response in favor of the tumor growth due to pro-inflammatory response in TME [136]. As can be seen, used as an inducer of ICD, RT is one of the best candidates to be used in combination with chemotherapy. However, it should be noted that the relevant effects of RT are in many cases based on the appropriate application at the right stage of tumor development, as well as the microenvironmental conditions. In the work reported by Frey et al. the importance of the type of ionization and its correlation with the evidence of immunosuppression derived from the administration of radiotherapy has been described in detail [137]. In this review, the authors also delineated the relevance of a personalized administration of radiotherapy taking into account the expression of immunocheckpoint molecules such as PD-L1, since there is evidence of upregulation upon some ionizing therapies of this immunosuppressive molecule.

#### 4.4.2. Photodynamic Therapy (PDT)

The use of light as anti-tumor therapy has been known since the beginning of the 20th century. The combination of this physical method with the administration of chemical compounds has been the key to the success of these agents [138]. In the context of ICD, the use of hypericin with PDT stands out. Hypericin (isolated from *Hypericum perforatum*) is a photoactive pigment with an anthraquinone structure forming a chromophore system. Light activation of these compounds causes ROS to be generated. This property together with the ability of this compound to accumulate in tissues has long been exploited as a selective cytotoxic treatment of tumor cells [139]. ROS production and ER stress induce immunogenic apoptosis and the corresponding DAMP signals. This combination has been considered one of the inducers of type II ICD due to its ability to directly target the formation of oxidative species in the ER lumen [12]. As an added value to this therapy, the ability of PDT-hypericin to inhibit the action of metalloproteases was reported. Metalloproteases such as matrix metallprotease-9 (MMP-9) play an important role in sculpting the tumor surroundings and play an important role in cell invasion and neovasculization, the effect of PDT-hypericin on these enzymes could also help to modulate the TME [140]. Like hypericin, there are other photosensitizers (PS) that have a potential to induce the formation of intracellular ROS, such as photofrin, 5-aminolevulinic acid, G-chlorin or rose Bengal [18]. More recently, bacteriochrolin macrocycles has emerged as novel PS, some of which can enable reaching deeper lesions. Two of these bacteriochlorin macrocycles, padeliporfin, and redaporfin are currently undergoing clinical trials [141].

The drawbacks of PDT derive from the hypoxia situation that usually occurs in TME and that causes decreased ROS formation [142]. One of the recent approaches to circumvent this problem is to design nanocarriers. For example, Li et al. reported the design of nanospheres containing ROS inducer peptides. Combining PDT and photothermal therapies, they have described T cell recruitment and cytokine release that correlated with DAMP signaling [143]. Another recent work from Yang and collaborators also employs nanotechnology to include peptides in nanovesicles for enhancement of ICD mediated by PTD [144].

Effects of PDT are also exhibited in a dose-dependent manner. As is the case for the other ICD inducers mentioned previously in this work, administration of low doses is required for an optimal immunogenic response. Using doses 10-fold lower than usually used for the cytotoxic effect, Doix and colleagues described the same CD8+ tumor recruitment [145]. These authors suggested that only the release of DAMPs is enough for creating a pro-inflammatory TME and for the activation of immunogenic response [145]. The possibility of combining PDT with different PS and other anti-tumoral drugs in a single schema makes this therapeutic strategy one of the most versatile approaches for ICD induction.

## 5. Monitoring ICD in TME

The development of ICD as a pharmacological strategy requires the correct detection of its indicators in vitro and in vivo. DAMPs are well-characterized biomarkers that, as explained above, play a fundamental role in ICD. In addition, in ICD, they become important when they can be released from the cell and reach the receptor that triggers the immune system’s inflammatory response.

CRT was one of the first DAMPs to be associated with ICD [19]. Exposure of CRT in the membrane is crucial for triggering the phagocytic response. Flow cytometry is a very affordable technique [149] that has been used to measure ecto-CRT in cell-response in the preclinical analysis [150] or in clinical practice [151]. Using the corresponding antibody, immunohistochemistry (IHC) can represent a good alternative method for monitoring patients’ progression and prognosis by CRT [152]. Engineering techniques such as transfections can also be used for more extensive monitoring of CRT function in vitro, although they are difficult to apply in clinical practice [80,153]. For the released DAMPs, it is essential to study the supernatants to verify their function and relevance in ICD since both ATP and HMGB1 have their own functions in the intracellular milieu. Antibody related techniques (ELISA or IHC) or specific kits are widely employed for measurement of HMGB1 [154,155]. However, different oxidized forms of HMGB1 or the other isoforms can lead to false positives. In this regard, the use of mass spectrometry proves to be the most appropriate method to distinguish among the different HMGB1 analogs [156].

**Figure 9:**
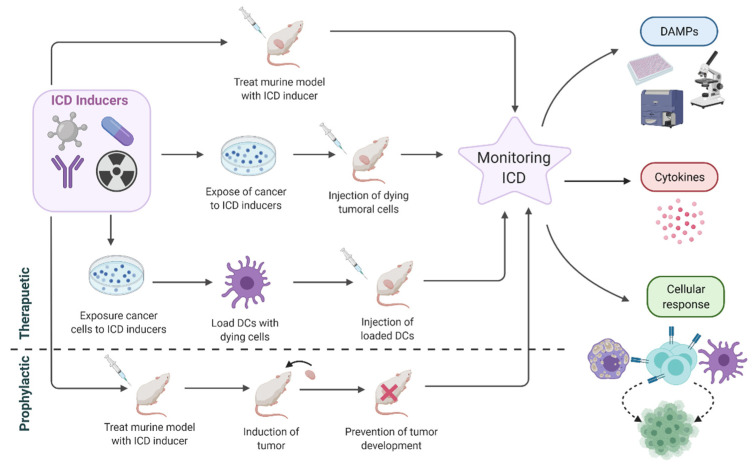
Schematic protocols to monitor ICD in TME.

ATP is a simpler chemical molecule than HGMB1. Luciferase-based methodologies are useful tools in the measurement of extracellular ATP as well as in the monitoring of dynamic changes to ADP or AMP [157]. Trafficking ATP vesicle can be followed by live cell imaging using fluorescent markers such as quinacrine [158]. Determination of efficacy of ICD inducers should be accompanied by a characterization of the induced immune response while DAMPs levels are also correlated (Figure 9). 

Intracellular pathways inducing ICD can be delimited using only tumoral cell culture as shown in the report of Giglio and collaborators. In this case, extensive description of DAMPs was shown by western blot, immunofluorescence and luciferin-based ATP assay, together with an ER stress signaling characterization on melanoma cell lines [159].

ICD inducers have been tested as therapeutics or prophylactic agents (Figure 9). An objective approach to evaluate an ICD inducer would be the administration of the drug to the induced tumor in mice and the monitoring of the tumor progression [23]. However, the induction of immunogenic activity of candidate ICD inducers can be shown when the cells pretreated with ICD inducers are administered as vaccines [108]. This model is used broadly in preclinical models and requires pretreatment with ICD inducers. An interesting approach was recently carried out by Geng and collaborators where doxorubicin was used as ICD inducer. In this case, the drug played the role of an adjuvant in the administration of a cancer vaccine, promoting the most favorable TME for increasing the prophylactic affect [160]. A well-conducted protocol for the success of this kind of therapies was detailed in the work of Pozzi and collaborators to prove cetuximab’s ICD induction. In this case, murine CT26 cell (colorectal cancer) were transfected with the human gene for EGFR and treated with cetuximab and a non-ICD inducing chemotherapy regimen. A high percentage of survival (90%) was observed in mice when they were vaccinated with cells that had been treated with cetuximab-chemotherapy combination, and then challenged by parental cell line without human EGFR expression [122]. ICD inducers can be used to obtain DC-based immunotherapy and can enhance immunogenicity and HLA-peptide presentation [161]. In this regard, DCs can be loaded with tumor cell lysate as in the work of Chen and collaborators where shikonin was used as an ICD inducer [73]. These preclinical approaches are followed by a complete characterization of DAMPs, cytokine and cellular responses in TME as was discussed above in the characterization if the different ICD inducers detailed.

## 6. Perspectives for ICD in TME

**Figure 10:**
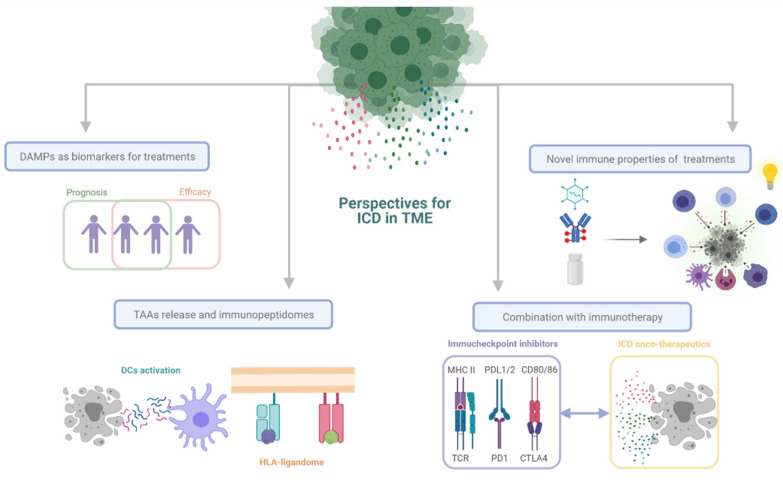
Different approaches for ICD in TME.

As we explained above, ICD intends to promote DC activation and increase the recruitment of T cells in the TME to assist in tumor remission. However, ICD can go beyond its role in activating the immune system to become a versatile tool in the treatment of cancer (Figure 10). 

The change of concept regarding the consequences of cell death, and the characterization of DAMPs within ICD not only raises the possibility of designing therapeutics based on these concepts but also the use of these two facets as biomarkers of prognosis. CRT exposure in drug administration correlates with good treatment response in patients with colorectal cancer, liver metastases or ovarian carcinoma [152,162]. In the same manner, HMGB1 and their associated receptors, and ATP release has been identified as markers for good prognosis as a probable indicator of favorable immunomodulation in TME [163,164]. Similarly, the use of ICD inducers and the study of DAMP levels in patients can help explain treatment-associated resistance or the success. In a report measuring ATP concentration in extracellular milieu, it was possible to correlate doxorubicin activity with the concentration of this DAMP [165]. Another report with more than 1000 patients determined the success of the use of anthracycline as adjuvant thanks to their capacity of inducing ICD. Low levels of HMGB1 were attributed to the failure in treatment [166]. Approaching the topic of biomarkers from another point of view, ICD and its correlation with the DAMPs also has a bearing on the efficacy of treatments. In this context, several approaches have been reported. Levels of HMGB1 has been linked to the efficacy of treatment in patients with breast cancer with better efficiency than monitoring other cancer biomarkers such as CA 15-3 and CEA [167] Accordingly, Rapoport and Anderson have written an interesting review on the association between ICD and treatment success, where they address not only DAMPs but also other immune cell components that may help in predicting the success of treatments [168].

As described in this work, the gold standard for ICD inducers consist of immuno-modulation of TME to promote tumor regression by restoring the activation of anti-tumor T cells. Besides, the “eat-me” or “find me” signals can appear because of the mechanism of action of the agent. These signals are extremely important for the recruitment of APCs and antigen presentation. All these characteristics promote the search for new molecules or anti-tumor agents that can induce ICD. Additionally, the use of novel techniques for the description of the mechanisms involved in cell death may provide the key for uncovering novel ICD inducers [169,170]. It is essential to point out that some ICD inducers require lower doses than usual to obtain the optimal immunomodulatory effect [145]. With this premise, new perspectives are opened for those compounds that did not achieve the standards to reach the clinic as anti-tumoral drugs. Natural products are also being targeted for the development of new ICD therapies [171]. It is difficult to establish a structure-activity relationship of ICD-inducing compounds. There is not even a defined biological target other than the development of targeted ER stress. Nevertheless, the versatility of structures and pharmacological mechanisms that can be found in these compounds makes the approach of high-throughput screening for immunological properties very appropriate [172].

There are guidelines for describing parameters to define whether an agent can be considered a bona fide inducer or whether its ICD is dependent on direct action on the ER [173]. However, a more detailed study of how antigenic presentation influences ICD is lacking.

More knowledge on the immunopeptidome would be useful for fine-tuning cancer vaccines, including the ones that could be produced by ICD inducers. Profiling epitopes that are presented upon ICD induction would shed some light on the mechanism of action of ICD and would provide insights to optimize this process for the desired effects. Bearing in mind this idea, more efforts in decoding immunopeptidomes should be invested as it was reported as resistance mechanism for other immunotherapies [174]. Human Immunopeptidome Project (HIPP) has been recently launched with the aim of providing a complete map of the human immunopeptidome and developing a robust and reproducible machinery that will be useful in translational clinical research [174]. Deciphering MHC-I and II loaded peptides is relevant as this can provide specific and individual information for personalized medicine. Despite the great advances in the knowledge and prediction of HLA-bound ligands, it remains a challenge because the antigens of interest currently represent a very small fraction of HLA-ligandome. Thanks to the current strategies and tools, we are improving epitope prediction and our understanding of epitope processing and presentation for clinical application [175].

ICDs and DAMPs can be used beyond their use as treatment, as neoadjuvant in therapeutics. These two variables have a high and specific prognostic value both in the search for treatment success and in the predictive value of disease remission [55]. The presence and release of DAMPs are closely related to the success of immunotherapy treatments since they provide an ideal TME for the immune response. This fact together with its therapeutic potential make ICD a versatile tool to be used as a molecular basis in the field of basic research to serve as a biomarker from bench to bedside [176].

## 7. Conclusions

Numerous efforts are being made to find the ultimate therapy against cancer. However, the current state of research in this field shows that there is no single treatment to kill all the tumor cells. The challenges of therapy design and development are many, one of which is the hostile conditions of the TME. Immunosuppression as well as other biochemical conditions make it extremely difficult for the drugs or therapeutic agents to reach the tumor cell or act effectively. These facts generate subsequent problems in pharmacodynamics and drug resistance. For this reason, ICD is a useful alternative to modulate the TME conditions in a rational and efficient manner.

As described in this work, there are currently different approved therapies with proven clinical evidence that, in addition to their anti-tumor effect, have the effect of triggering an immune response against the tumour. Harnessing “programmable death” is emerging as a new tool to promote tumour regression from two different points of view. First, with cell death intrinsic to the drugs, and second, by taking advantage of the immunogenic effects derived from cell death itself.

ICD plays an important role in immunotherapy and in the improvement of cancer therapy. Restoring immunosurveillance against the tumours may be the key to increase effectiveness of current treatments. ER stress and DAMP signalling are the most prominent features that signal the immunogenic modulation of the tumour environment and there is a great variability of therapeutic approaches to promote this. Further in-depth study of each of the features of the ICD can lead to great advances of this strategy in research and clinical practice.

## Figures and Tables

**Table 1:** Overview of major hallmarks of ICD inducers based on their molecular target and DAMP response-mediated.

Treatment	Molecular Target	DAMP Released				References
		CRT	HMGB1	ATP	Others	
Type I inducers						
Anthracylcines	DNA intercalant Topoisomerase-II inhibitors	X	X	X	HSPs IFN	[19,63,64,65,66]
Shikonin	PKMa	X			HSPs	[146]
Cardiac Glycosides	Na^+^/K^+^ ATPase	X	X	X		[80]
Paclitaxel	Tubulin	X	X		IFN	[85,147]
Oxaliplatin	Alkylating agent	X	X	X	IFN	[91]
Cyclophosphamide		X	X	X	IFN	[100]
Bortezomib	26s++ proteasome	X	X		HSPs IFN	[108,109]
Vorinostat	HDAC inhibitor	X	X	X		[114,115]
Monoclonal antibodies	EGFR Receptor	X			HSPs	[119,122]
Radiotherapy	DNA damage	X	X	X	HSPs IFN	[135]
Type II inducers						
Oncolytic viruses	ER stress	X	X	X	IFN	[126,148]
PDT-hypericin	ER stress	X	X	X		[143]

## Data Availability

Not applicable.

## Abbreviations

ICD: Immunogenic cell death. DAMPs: damage-associated molecular patterns. TME: tumor microenvironment. APCs: antigen-presenting cells. TAAs: tumor-associated antigens. ER: endoplasmic reticulum. IFN1: type I IFN. eIF2A: eukaryotic translation initiation factor 2A. UPR: unfolded protein response. CRT: Calreticulin. HMGB1: high mobility group box 1 protein. RAGE: receptor for advanced glycation endproducts. LAMP: lysosomal-associated membrane protein. HSPs: Heat shock proteins. ANXA1: annexin A1. FPR1: formyl peptide receptor 1. CG: Cardiac Glycosides. PTX: Paclitaxel. OX: Oxaliplatin. CPA: Cyclophosphamide. HDACi: histone deacetylase inhibitor. OV: Oncolytic viruses. RT: Radiotherapy. PDT: Photodynamic Therapy. MDSCs: Myeloid-derived suppressor cells.

## References

[1] Hanahan D., Weinberg R.A. (2000). The hallmarks of cancer. Cell.

[2] Hanahan D., Weinberg R.A. (2011). Hallmarks of Cancer: The Next Generation. Cell.

[3] Correia A.L., Bissell M.J. (2012). The tumor microenvironment is a dominant force in multidrug resistance. Drug Resist. Updates.

[4] Kersten K., Salvagno C., de Visser K.E. (2015). Exploiting the immunomodulatory properties of chemotherapeutic drugs to improve the success of cancer immunotherapy. Front. Immunol..

[5] Schreiber R.D., Old L.J., Smyth M.J. (2011). Cancer Immunoediting: Integrating Immunity’s Roles in Cancer Suppression and Promotion. Science.

[6] O’Sullivan T., Saddawi-Konefka R., Vermi W., Koebel C.M., Arthur C., White J.M., Uppaluri R., Andrews D.M., Ngiow S.F., Teng M.W.L. (2012). Cancer immunoediting by the innate immune system in the absence of adaptive immunity. J. Exp. Med..

[7] Tang S., Ning Q., Yang L., Mo Z., Tang S. (2020). Mechanisms of immune escape in the cancer immune cycle. Int. Immunopharmacol..

[8] Wang D., Yang L.H., Zhang P., LaBaer J., Hermjakob H., Li D., Yu X.B. (2017). AAgAtlas 1.0: A human autoantigen database. Nucleic Acids Res..

[9] Yarchoan M., Johnson B.A., Lutz E.R., Laheru D.A., Jaffee E.M. (2017). Targeting neoantigens to augment antitumour immunity. Nat. Rev. Cancer.

[10] De Guillebon E., Dardenne A., Saldmann A., Seguier S., Tran T., Paolini L., Lebbe C., Tartour E. (2020). Beyond the concept of cold and hot tumors for the development of novel predictive biomarkers and the rational design of immunotherapy combination. Int. J. Cancer.

[11] Acebes-Fernandez V., Landeira-Vinuela A., Juanes-Velasco P., Hernandez A.-P., Otazo-Perez A., Manzano-Roman R., Gongora R., Fuentes M. (2020). Nanomedicine and Onco-Immunotherapy: From the Bench to Bedside to Biomarkers. Nanomaterials.

[12] Krysko D.V., Garg A.D., Kaczmarek A., Krysko O., Agostinis P., Vandenabeele P. (2012). Immunogenic cell death and DAMPs in cancer therapy. Nat. Rev. Cancer.

[13] Galluzzi L., Vitale I., Aaronson S.A., Abrams J.M., Adam D., Agostinis P., Alnemri E.S., Altucci L., Amelio I., Andrews D.W. (2018). Molecular mechanisms of cell death: Recommendations of the Nomenclature Committee on Cell Death 2018. Cell Death Differ..

[14] Green D.R., Ferguson T., Zitvogel L., Kroemer G. (2009). Immunogenic and tolerogenic cell death. Nat. Rev. Immunol..

[15] Galluzzi L., Buque A., Kepp O., Zitvogel L., Kroemer G. (2017). Immunogenic cell death in cancer and infectious disease. Nat. Rev. Immunol..

[16] Wang H., Sun L., Su L., Rizo J., Liu L., Wang L.-F., Wang F.-S., Wang X. (2014). Mixed Lineage Kinase Domain-like Protein MLKL Causes Necrotic Membrane Disruption upon Phosphorylation by RIP3. Mol. Cell.

[17] Kurokawa T., Oelke M., Mackensen A. (2001). Induction and clonal expansion of tumor-specific cytotoxic T lymphocytes from renal cell carcinoma patients after stimulation with autologous dendritic cells loaded with tumor cells. Int. J. Cancer.

[18] Dudek A.M., Garg A.D., Krysko D.V., De Ruysscher D., Agostinis P. (2013). Inducers of immunogenic cancer cell death. Cytokine Growth Factor Rev..

[19] Obeid M., Tesniere A., Ghiringhelli F., Fimia G.M., Apetoh L., Perfettini J.L., Castedo M., Mignot G., Panaretakis T., Casares N. (2007). Calreticulin exposure dictates the immunogenicity of cancer cell death. Nat. Med..

[20] Garg A.D., Nowis D., Golab J., Vandenabeele P., Krysko D.V., Agostinis P. (2010). Immunogenic cell death, DAMPs and anticancer therapeutics: An emerging amalgamation. Biochim. Biophys. Acta Rev. Cancer.

[21] Gold L.I., Eggleton P., Sweetwyne M.T., Van Duyn L.B., Greives M.R., Naylor S.-M., Michalak M., Murphy-Ullrich J.E. (2010). Calreticulin: Non-endoplasmic reticulum functions in physiology and disease. FASEB J..

[22] Mesaeli N., Phillipson C. (2004). Impaired p53 expression, function, and nuclear localization in calreticulin-deficient cells. Mol. Biol. Cell.

[23] Tesniere A., Schlemmer F., Boige V., Kepp O., Martins I., Ghiringhelli F., Aymeric L., Michaud M., Apetoh L., Barault L. (2010). Immunogenic death of colon cancer cells treated with oxaliplatin. Oncogene.

[24] Panaretakis T., Kepp O., Brockmeier U., Tesniere A., Bjorklund A.-C., Chapman D.C., Durchschlag M., Joza N., Pierron G., van Endert P. (2009). Mechanisms of pre-apoptotic calreticulin exposure in immunogenic cell death. EMBO J..

[25] Kroemer G., Galluzzi L., Kepp O., Zitvogel L. (2013). Immunogenic Cell Death in Cancer Therapy. Annu. Rev. Immunol..

[26] Ghiringhelli F., Apetoh L., Tesniere A., Aymeric L., Ma Y., Ortiz C., Vermaelen K., Panaretakis T., Mignot G., Ullrich E. (2009). Activation of the NLRP3 inflammasome in dendritic cells induces IL-1 beta-dependent adaptive immunity against tumors. Nat. Med..

[27] Bezu L., Sauvat A., Humeau J., Gomes-da-Silva L.C., Iribarren K., Forveille S., Garcia P., Zhao L., Liu P., Zitvogel L. (2018). eIF2 alpha phosphorylation is pathognomonic for immunogenic cell death. Cell Death Differ..

[28] Sukkurwala A.Q., Martins I., Wang Y., Schlemmer F., Ruckenstuhl C., Durchschlag M., Michaud M., Senovilla L., Sistigu A., Ma Y. (2014). Immunogenic calreticulin exposure occurs through a phylogenetically conserved stress pathway involving the chemokine CXCL8. Cell Death Differ..

[29] Tarr J.M., Young P.J., Morse R., Shaw D.J., Haigh R., Petrov P.G., Johnson S.J., Winyard P.G., Eggleton P. (2010). A Mechanism of Release of Calreticulin from Cells During Apoptosis. J. Mol. Biol..

[30] Hernandez C., Huebener P., Schwabe R.F. (2016). Damage-associated molecular patterns in cancer: A double-edged sword. Oncogene.

[31] Zeng G., Aldridge M.E., Tian X., Seiler D., Zhang X., Jin Y., Rao J., Li W., Chen D., Langford M.P. (2006). Dendritic cell surface calreticulin is a receptor for NY-ESO-1: Direct interactions between tumor-associated antigen and the innate immune system. J. Immunol..

[32] Martins-Teixeira M.B., Carvalho I. (2020). Antitumour Anthracyclines: Progress and Perspectives. Chemmedchem.

[33] Li C., Sun H., Wei W., Liu Q., Wang Y., Zhang Y., Lian F., Liu F., Li C., Ying K. (2020). Mitoxantrone triggers immunogenic prostate cancer cell death via p53-dependent PERK expression. Cell. Oncol..

[34] Zhu H., Shan Y., Ge K., Lu J., Kong W., Jia C. (2020). Oxaliplatin induces immunogenic cell death in hepatocellular carcinoma cells and synergizes with immune checkpoint blockade therapy. Cell. Oncol..

[35] Walle T., Martinez Monge R., Cerwenka A., Ajona D., Melero I., Lecanda F. (2018). Radiation effects on antitumor immune responses: Current perspectives and challenges. Ther. Adv. Med. Oncol..

[36] Nogueira-Machado J.A., de Oliveira Volpe C.M., Veloso C.A., Chaves M.M. (2011). HMGB1, TLR and RAGE: A functional tripod that leads to diabetic inflammation. Expert Opin. Ther. Targets.

[37] Hreggvidsdottir H.S., Lundberg A.M., Aveberger A.-C., Klevenvall L., Andersson U., Harris H.E. (2012). High Mobility Group Box Protein 1 (HMGB1)-Partner Molecule Complexes Enhance Cytokine Production by Signaling Through the Partner Molecule Receptor. Mol. Med..

[38] Palumbo R., Sampaolesi M., De Marchis F., Tonlorenzi R., Colombetti S., Mondino A., Cossu G., Bianchi M.E. (2004). Extracellular HMGB1, a signal of tissue damage, induces mesoangioblast migration and proliferation. J. Cell Biol..

[39] Park J.S., Svetkauskaite D., He Q.B., Kim J.Y., Strassheim D., Ishizaka A., Abraham E. (2004). Involvement of toll-like receptors 2 and 4 in cellular activation by high mobility group box 1 protein. J. Biol. Chem..

[40] Bianchi M.E., Crippa M.P., Manfredi A.A., Mezzapelle R., Querini P.R., Venereau E. (2017). High-mobility group box 1 protein orchestrates responses to tissue damage via inflammation, innate and adaptive immunity, and tissue repair. Immunol. Rev..

[41] Yang H., Hreggvidsdottir H.S., Palmblad K., Wang H., Ochani M., Li J., Lu B., Chavan S., Rosas-Ballina M., Al-Abed Y. (2010). A critical cysteine is required for HMGB1 binding to Toll-like receptor 4 and activation of macrophage cytokine release. Proc. Natl. Acad. Sci. Am..

[42] Laura Policastro L., Laura Ibanez I., Notcovich C., Alicia Duran H., Luis Podhajcer O. (2013). The Tumor Microenvironment: Characterization, Redox Considerations, and Novel Approaches for Reactive Oxygen Species-Targeted Gene Therapy. Antioxid. Redox Signal..

[43] Yang H., Antoine D.J., Andersson U., Tracey K.J. (2013). The many faces of HMGB1: Molecular structure-functional activity in inflammation, apoptosis, and chemotaxis. J. Leukoc. Biol..

[44] Gordon J.L. (1986). Extracellular ATP: Effects, sources and fate. Biochem. J..

[45] Elliott M.R., Chekeni F.B., Trampont P.C., Lazarowski E.R., Kadl A., Walk S.F., Park D., Woodson R.I., Ostankovich M., Sharma P. (2009). Nucleotides released by apoptotic cells act as a find-me signal to promote phagocytic clearance. Nature.

[46] Di Virgilio F., Adinolfi E. (2017). Extracellular purines, purinergic receptors and tumor growth. Oncogene.

[47] la Sala A., Ferrari D., Corinti S., Cavani A., Di Virgilio F., Girolomoni G. (2001). Extracellular ATP induces a distorted maturation of dendritic cells and inhibits their capacity to initiate Th1 responses. J. Immunol..

[48] Beavis P.A., Stagg J., Darcy P.K., Smyth M.J. (2012). CD73: A potent suppressor of antitumor immune responses. Trends Immunol..

[49] Carini R., Trincheri N.F., Alchera E., De Cesaris M.G., Castino R., Splendore R., Albano E., Isidoro C. (2006). PI3K-dependent lysosome exocytosis in nitric oxide-preconditioned hepatocytes. Free Radic. Biol. Med..

[50] Chekeni F.B., Elliott M.R., Sandilos J.K., Walk S.F., Kinchen J.M., Lazarowski E.R., Armstrong A.J., Penuela S., Laird D.W., Salvesen G.S. (2010). Pannexin 1 channels mediate ‘find-me’ signal release and membrane permeability during apoptosis. Nature.

[51] Wang Y., Martins I., Ma Y., Kepp O., Galluzzi L., Kroemer G. (2013). Autophagy-dependent ATP release from dying cells via lysosomal exocytosis. Autophagy.

[52] Lanneau D., Brunet M., Frisan E., Solary E., Fontenay M., Garrido C. (2008). Heat shock proteins: Essential proteins for apoptosis regulation. J. Cell. Mol. Med..

[53] Melcher A., Todryk S., Hardwick N., Ford M., Jacobson M., Vile R.G. (1998). Tumor immunogenicity is determined by the mechanism of cell death via induction of heat shock protein expression. Nat. Med..

[54] Spisek R., Dhodapkar M.V. (2007). Towards a better way to die with chemotherapy—Role of heat shock protein exposure on dying tumor cells. Cell Cycle.

[55] Fucikova J., Moserova I., Urbanova L., Bezu L., Kepp O., Cremer I., Salek C., Strnad P., Kroemer G., Galluzzi L. (2015). Prognostic and predictive value of DAMPs and DAMP-associated processes in cancer. Front. Immunol..

[56] Vacchelli E., Ma Y., Baracco E.E., Sistigu A., Enot D.P., Pietrocola F., Yang H., Adjemian S., Chaba K., Semeraro M. (2015). Chemotherapy-induced antitumor immunity requires formyl peptide receptor 1. Science.

[57] Ivashkiv L.B., Donlin L.T. (2014). Regulation of type I interferon responses. Nat. Rev. Immunol..

[58] Sistigu A., Yamazaki T., Vacchelli E., Chaba K., Enot D.P., Adam J., Vitale I., Goubar A., Baracco E.E., Remedios C. (2014). Cancer cell-autonomous contribution of type I interferon signaling to the efficacy of chemotherapy. Nat. Med..

[59] Aaes T.L., Vandenabeele P. (2020). The intrinsic immunogenic properties of cancer cell lines, immunogenic cell death, and how these influence host antitumor immune responses. Cell Death Differ..

[60] Garg A.D., More S., Rufo N., Mece O., Sassano M.L., Agostinis P., Zitvogel L., Kroemer G., Galluzzi L. (2017). Trial watch: Immunogenic cell death induction by anticancer chemotherapeutics. Oncoimmunology.

[61] Talib W.H., Alsalahat I., Daoud S., Abutayeh R.F., Mahmod A.I. (2020). Plant-Derived Natural Products in Cancer Research: Extraction, Mechanism of Action, and Drug Formulation. Molecules.

[62] Minotti G., Menna P., Salvatorelli E., Cairo G., Gianni L. (2004). Anthracyclines: Molecular advances and pharmacologic developments in antitumor activity and cardiotoxicity. Pharmacol. Rev..

[63] Casares N., Pequignot M.O., Tesniere A., Ghiringhelli F., Roux S., Chaput N., Schmitt E., Hamai A., Hervas-Stubbs S., Obeid M. (2005). Caspase-dependent immunogenicity of doxorubicin-induced tumor cell death. J. Exp. Med..

[64] Fucikova J., Kralikova P., Fialova A., Brtnicky T., Rob L., Bartunkova J., Spisek R. (2011). Human Tumor Cells Killed by Anthracyclines Induce a Tumor-Specific Immune Response. Cancer Res..

[65] Aymeric L., Apetoh L., Ghiringhelli F., Tesniere A., Martins I., Kroemer G., Smyth M.J., Zitvogel L. (2010). Tumor Cell Death and ATP Release Prime Dendritic Cells and Efficient Anticancer Immunity. Cancer Res..

[66] Ma Y., Adjemian S., Mattarollo S.R., Yamazaki T., Aymeric L., Yang H., Catani J.P.P., Hannani D., Duret H., Steegh K. (2013). Anticancer Chemotherapy-Induced Intratumoral Recruitment and Differentiation of Antigen-Presenting Cells. Immunity.

[67] Ma Y., Aymeric L., Locher C., Mattarollo S.R., Delahaye N.F., Pereira P., Boucontet L., Apetoh L., Ghiringhelli F., Casares N. (2011). Contribution of IL-17-producing gamma delta T cells to the efficacy of anticancer chemotherapy. J. Exp. Med..

[68] Inoue S., Setoyama Y., Odaka A. (2014). Doxorubicin treatment induces tumor cell death followed by immunomodulation in a murine neuroblastoma model. Exp. Ther. Med..

[69] Castoldi F., Vacchelli E., Zitvogel L., Maiuri M.C., Pietrocola F., Kroemer G. (2019). Systemic autophagy in the therapeutic response to anthracycline-based chemotherapy. Oncoimmunology.

[70] Vanmeerbeek I., Sprooten J., De Ruysscher D., Tejpar S., Vandenberghe P., Fucikova J., Spisek R., Zitvogel L., Kroemer G., Galluzzi L. (2020). Trial watch: Chemotherapy-induced immunogenic cell death in immuno-oncology. Oncoimmunology.

[71] Chen X., Yang L., Oppenheim J.J., Howard O.M.Z. (2002). Cellular pharmacology studies of shikonin derivatives. Phytother. Res..

[72] Chang I.C., Huang Y.-J., Chiang T.-I., Yeh C.-W., Hsu L.-S. (2010). Shikonin Induces Apoptosis through Reactive Oxygen Species/Extracellular Signal-Regulated Kinase Pathway in Osteosarcoma Cells. Biol. Pharm. Bull..

[73] Chen H.M., Wang P.H., Chen S.S., Wen C.C., Chen Y.H., Yang W.C., Yang N.S. (2012). Shikonin induces immunogenic cell death in tumor cells and enhances dendritic cell-based cancer vaccine. Cancer Immunol. Immunother..

[74] Lin S.Y., Hsieh S.Y., Fan Y.T., Wei W.C., Hsiao P.W., Tsai D.H., Wu T.S., Yang N.S. (2018). Necroptosis promotes autophagy-dependent upregulation of DAMP and results in immunosurveillance. Autophagy.

[75] Lin T.J., Lin H.T., Chang W.T., Mitapalli S.P., Hsiao P.W., Yin S.Y., Yang N.S. (2015). Shikonin-enhanced cell immunogenicity of tumor vaccine is mediated by the differential effects of DAMP components. Mol. Cancer.

[76] Chen Y., Gao Y., Yi X., Zhang J., Chen Z., Wu Y. (2020). Integration of Transcriptomics and Metabolomics Reveals the Antitumor Mechanism Underlying Shikonin in Colon Cancer. Front. Pharmacol..

[77] Stenkvist B., Bengtsson E., Eriksson O., Holmquist J., Nordin B., Westmannaeser S., Eklund G. (1979). CARDIAC-GLYCOSIDES AND BREAST-CANCER. Lancet.

[78] Winnicka K., Bielawski K., Bielawska A. (2006). Cardiac glycosides in cancer research and cancer therapy. Acta Pol. Pharm..

[79] Prassas I., Diamandis E.P. (2008). Novel therapeutic applications of cardiac glycosides. Nat. Rev. Drug Discov..

[80] Menger L., Vacchelli E., Adjemian S., Martins I., Ma Y.T., Shen S.S., Yamazaki T., Sukkurwala A.Q., Michaud M., Mignot G. (2012). Cardiac Glycosides Exert Anticancer Effects by Inducing Immunogenic Cell Death. Sci. Transl. Med..

[81] Menger L., Vacchelli E., Kepp O., Eggermont A., Tartour E., Zitvogel L., Kroemer G., Galluzzi L. (2013). Trial watch Cardiac glycosides and cancer therapy. Oncoimmunology.

[82] Sukkurwala A.Q., Adjemian S., Senovilla L., Michaud M., Spaggiari S., Vacchelli E., Baracco E.E., Galluzzi L., Zitvogel L., Kepp O. (2014). Screening of novel immunogenic cell death inducers within the NCI Mechanistic Diversity Set. Oncoimmunology.

[83] Yuan B., He J., Kisoh K., Hayashi H., Tanaka S., Si N., Zhao H.Y., Hirano T., Bian B.L., Takagi N. (2016). Effects of active bufadienolide compounds on human cancer cells and CD4(+)CD25(+)Foxp3(+) regulatory T cells in mitogen-activated human peripheral blood mononuclear cells. Oncol. Rep..

[84] Pol J., Vacchelli E., Aranda F., Castoldi F., Eggermont A., Cremer I., Sautes-Fridman C., Fucikova J., Galon J., Spisek R. (2015). Trial Watch: Immunogenic cell death inducers for anticancer chemotherapy. Oncoimmunology.

[85] Senovilla L., Vitale I., Martins I., Tailler M., Pailleret C., Michaud M., Galluzzi L., Adjemian S., Kepp O., Niso-Santano M. (2012). An Immunosurveillance Mechanism Controls Cancer Cell Ploidy. Science.

[86] Huang B., Sikorski R., Kirn D.H., Thorne S.H. (2011). Synergistic anti-tumor effects between oncolytic vaccinia virus and paclitaxel are mediated by the IFN response and HMGB1. Gene Ther..

[87] Pfannenstiel L.W., Lam S.S.K., Emens L.A., Jaffee E.M., Armstrong T.D. (2010). Paclitaxel enhances early dendritic cell maturation and function through TLR4 signaling in mice. Cell. Immunol..

[88] Lau T.S., Chan L.K.Y., Man G.C.W., Wong C.H., Lee J.H.S., Yim S.F., Cheung T.H., McNeish I.A., Kwong J. (2020). Paclitaxel Induces Immunogenic Cell Death in Ovarian Cancer via TLR4/IKK2/SNARE-Dependent Exocytosis. Cancer Immunol. Res..

[89] Martinez-Balibrea E., Martinez-Cardus A., Gines A., Ruiz de Porras V., Moutinho C., Layos L., Luis Manzano J., Buges C., Bystrup S., Esteller M. (2015). Tumor-Related Molecular Mechanisms of Oxaliplatin Resistance. Mol. Cancer Ther..

[90] Martins I., Tesniere A., Kepp O., Michaud M., Schlemmer F., Senovilla L., Seror C., Metivier D., Perfettini J.-L., Zitvogel L. (2009). Chemotherapy induces ATP release from tumor cells. Cell Cycle.

[91] Apetoh L., Ghiringhelli F., Tesniere A., Obeid M., Ortiz C., Criollo A., Mignot G., Maiuri M.C., Ullrich E., Saulnier P. (2007). Toll-like receptor 4-dependent contribution of the immune system to anticancer chemotherapy and radiotherapy. Nat. Med..

[92] Sato E., Olson S.H., Ahn J., Bundy B., Nishikawa H., Qian F., Jungbluth A.A., Frosina D., Gnjatic S., Ambrosone C. (2005). Intraepithelial CD8(+) tumor-infiltrating lymphocytes and a high CD8(+)/regulatory T cell ratio are associated with favorable prognosis in ovarian cancer. Proc. Natl. Acad. Sci. USA.

[93] Gou H.-F., Zhou L., Huang J., Chen X.-C. (2018). Intraperitoneal oxaliplatin administration inhibits the tumor immunosuppressive microenvironment in an abdominal implantation model of colon cancer. Mol. Med. Rep..

[94] Roberts N.B., Alqazzaz A., Hwang J.R., Qi X., Keegan A.D., Kim A.J., Winkles J.A., Woodworth G.F. (2018). Oxaliplatin disrupts pathological features of glioma cells and associated macrophages independent of apoptosis induction. J. Neuro-Oncol..

[95] Park S.-J., Ye W., Xiao R., Silvin C., Padget M., Hodge J.W., Van Waes C., Schmitt N.C. (2019). Cisplatin and oxaliplatin induce similar immunogenic changes in preclinical models of head and neck cancer. Oral Oncol..

[96] Pasquier E., Kavallaris M., Andre N. (2010). Metronomic chemotherapy: New rationale for new directions. Nat. Rev. Clin. Oncol..

[97] Wang W., Wu L., Zhang J., Wu H., Han E., Guo Q. (2017). Chemoimmunotherapy by combining oxaliplatin with immune checkpoint blockades reduced tumor burden in colorectal cancer animal model. Biochem. Biophys. Res. Commun..

[98] Sun F.F., Cui L.J., Li T.T., Chen S.L., Song J.M., Li D.Z. (2019). Oxaliplatin induces immunogenic cells death and enhances therapeutic efficacy of checkpoint inhibitor in a model of murine lung carcinoma. J. Recept. Signal Transduct..

[99] Colvin O.M. (1999). An overview of cyclophosphamide development and clinical applications. Curr. Pharm. Des..

[100] Schiavoni G., Sistigu A., Valentini M., Mattei F., Sestili P., Spadaro F., Sanchez M., Lorenzi S., D’Urso M.T., Belardelli F. (2011). Cyclophosphamide Synergizes with Type I Interferons through Systemic Dendritic Cell Reactivation and Induction of Immunogenic Tumor Apoptosis. Cancer Res..

[101] Schiavoni G., Mattei F., Di Pucchio T., Santini S.M., Bracci L., Belardelli F., Proietti E. (2000). Cyclophosphamide induces type I interferon and augments the number of CD44(hi) T lymphocytes in mice: Implications for strategies of chemoimmunotherapy of cancer. Blood.

[102] Matar P., Rozados V.R., Gonzalez A.D., Dlugovitzky D.G., Bonfil R.D., Scharovsky O.G. (2000). Mechanism of antimetastatic immunopotentiation by low-dose cyclophosphamide. Eur. J. Cancer.

[103] Doloff J.C., Waxman D.J. (2015). Transcriptional profiling provides insights into metronomic cyclophosphamide-activated, innate immune-dependent regression of brain tumor xenografts. BMC Cancer.

[104] Ghiringhelli F., Menard C., Puig P.E., Ladoire S., Roux S., Martin F., Solary E., Le Cesne A., Zitvogel L., Chauffert B. (2007). Metronomic cyclophosphamide regimen selectively depletes CD4(+) CD25(+) regulatory T cells and restores T and NK effector functions in end stage cancer patients. Cancer Immunol. Immunother..

[105] Audia S., Nicolas A., Cathelin D., Larmonier N., Ferrand C., Foucher P., Fanton A., Bergoin E., Maynadie M., Arnould L. (2007). Increase of CD4(+)CD25(+) regulatory T cells in the peripheral blood of patients with metastatic carcinoma: A Phase I clinical trial using cyclophosphamide and immunotherapy to eliminate CD4(+)CD25(+) T lymphocytes. Clin. Exp. Immunol..

[106] Chen D., Frezza M., Schmitt S., Kanwar J., Dou Q.P. (2011). Bortezomib as the First Proteasome Inhibitor Anticancer Drug: Current Status and Future Perspectives. Curr. Cancer Drug Targets.

[107] Nawrocki S.T., Carew J.S., Dunner K., Boise L.H., Chiao P.J., Huang P., Abbruzzese J.L., McConkey D.J. (2005). Bortezomib inhibits PKR-like endoplasmic reticulum (ER) kinase and induces apoptosis via ER stress in human pancreatic cancer cells. Cancer Res..

[108] Spisek R., Charalambous A., Mazumder A., Vesole D.H., Jagannath S., Dhodapkar M.V. (2007). Bortezomib enhances dendritic cell (DC)-mediated induction of immunity to human myeloma via exposure of cell surface heat shock protein 90 on dying tumor cells: Therapeutic implications. Blood.

[109] Chang C.L., Hsu Y.T., Wu C.C., Yang Y.C., Wang C., Wu T.C., Hung C.F. (2012). Immune Mechanism of the Antitumor Effects Generated by Bortezomib. J. Immunol..

[110] Pellom S.T., Dudimah D.F., Thounaojam M.C., Uzhachenko R.V., Singhal A., Richmond A., Shanker A. (2017). Bortezomib augments lymphocyte stimulatory cytokine signaling in the tumor microenvironment to sustain CD8(+)T cell antitumor function. Oncotarget.

[111] Hontecillas-Prieto L., Flores-Campos R., Silver A., de Alava E., Hajji N., Garcia-Dominguez D.J. (2020). Synergistic Enhancement of Cancer Therapy Using HDAC Inhibitors: Opportunity for Clinical Trials. Front. Genet..

[112] Duvic M., Talpur R., Ni X., Zhang C., Hazarika P., Kelly C., Chiao J.H., Reilly J.F., Ricker J.L., Richon V.M. (2007). Phase 2 trial of oral vorinostat (suberoylanilide hydroxamic acid, SAHA) for refractory cutaneous T-cell lymphoma, (CTCL). Blood.

[113] Shakespear M.R., Halili M.A., Irvine K.M., Fairlie D.P., Sweet M.J. (2011). Histone deacetylases as regulators of inflammation and immunity. Trends Immunol..

[114] Sonnemann J., Gressmann S., Becker S., Wittig S., Schmudde M., Beck J.F. (2010). The histone deacetylase inhibitor vorinostat induces calreticulin exposure in childhood brain tumour cells in vitro. Cancer Chemother. Pharmacol..

[115] West A.C., Mattarollo S.R., Shortt J., Cluse L.A., Christiansen A.J., Smyth M.J., Johnstone R.W. (2013). An Intact Immune System Is Required for the Anticancer Activities of Histone Deacetylase Inhibitors. Cancer Res..

[116] Gray J.E., Saltos A., Tanvetyanon T., Haura E.B., Creelan B., Antonia S.J., Shafique M., Zheng H., Dai W.J., Saller J.J. (2019). Phase I/Ib Study of Pembrolizumab Plus Vorinostat in Advanced/Metastatic Non-Small Cell Lung Cancer. Clin. Cancer Res..

[117] Reichert J.M., Dhimolea E. (2012). The future of antibodies as cancer drugs. Drug Discov. Today.

[118] Garrido G., Sanchez B., Rodriguez H.M., Lorenzano P., Alonso D., Fernandez L.E. (2004). 7A7 MAb: A new tool for the pre-clinical evaluation of EGFR-based therapies. Hybrid. Hybridomics.

[119] Garrido G., Rabasa A., Sanchez B., Victoria Lopez M., Blanco R., Lopez A., Rosa Hernandez D., Perez R., Enrique Fernandez L. (2011). Induction of Immunogenic Apoptosis by Blockade of Epidermal Growth Factor Receptor Activation with a Specific Antibody. J. Immunol..

[120] Garrido G., Rabasa A., Garrido C., Lopez A., Chao L., Garcia-Lora A.M., Garrido F., Fernandez L.E., Sanchez B. (2014). Preclinical modeling of EGFR-specific antibody resistance: Oncogenic and immune-associated escape mechanisms. Oncogene.

[121] He X., Cruz J.L., Joseph S., Pett N., Chew H.Y., Tuong Z.K., Okano S., Kelly G., Veitch M., Simpson F. (2018). Characterization of 7A7, an anti-mouse EGFR monoclonal antibody proposed to be the mouse equivalent of cetuximab. Oncotarget.

[122] Pozzi C., Cuomo A., Spadoni I., Magni E., Silvola A., Conte A., Sigismund S., Ravenda P.S., Bonaldi T., Zampino M.G. (2016). The EGFR-specific antibody cetuximab combined with chemotherapy triggers immunogenic cell death. Nat. Med..

[123] Inoue Y., Hazama S., Suzuki N., Tokumitsu Y., Kanekiyo S., Tomochika S., Tsunedomi R., Tokuhisa Y., Iida M., Sakamoto K. (2017). Cetuximab strongly enhances immune cell infiltration into liver metastatic sites in colorectal cancer. Cancer Sci..

[124] Kirn D. (2001). Oncolytic virotherapy for cancer with the adenovirus dl1520 (Onyx-015): Results of Phase I and II trials. Expert Opin. Biol. Ther..

[125] Prestwich R.J., Harrington K.J., Pandha H.S., Vile R.G., Melcher A.A., Errington F. (2008). Oncolytic viruses: A novel form of immunotherapy. Expert Rev. Anticancer Ther..

[126] Takasu A., Masui A., Hamada M., Imai T., Iwai S., Yura Y. (2016). Immunogenic cell death by oncolytic herpes simplex virus type 1 in squamous cell carcinoma cells. Cancer Gene Ther..

[127] Heinrich B., Klein J., Delic M., Goepfert K., Engel V., Geberzahn L., Lusky M., Erbs P., Preville X., Moehler M. (2017). Immunogenicity of oncolytic vaccinia viruses JX-GFP and TG6002 in a human melanoma in vitro model: Studying immunogenic cell death, dendritic cell maturation and interaction with cytotoxic T lymphocytes. Oncotargets Ther..

[128] Ye T., Jiang K., Wei L.W., Barr M.P., Xu Q., Zhang G.R., Ding C., Meng S.S., Piao H.Z. (2018). Oncolytic Newcastle disease virus induces autophagy-dependent immunogenic cell death in lung cancer cells. Am. J. Cancer Res..

[129] Zhang B., Cheng P. (2020). Improving antitumor efficacy via combinatorial regimens of oncolytic virotherapy. Mol. Cancer.

[130] Institute N.C. Radiation Therapy to Treat Cancer. https://www.cancer.gov/about-cancer/treatment/types/radiation-therapy.

[131] Formenti S.C., Demaria S. (2009). Systemic effects of local radiotherapy. Lancet Oncol..

[132] Ishihara H., Tsuneoka K., Dimchev A.B., Shikita M. (1993). Induction of the expression of the interleukin-1 beta gene in mouse spleen by ionizing radiation. Radiat. Res..

[133] Reits E.A., Hodge J.W., Herberts C.A., Groothuis T.A., Chakraborty M., Wansley E.K., Camphausen K., Luiten R.M., de Ru A.H., Neijssen J. (2006). Radiation modulates the peptide repertoire, enhances MHC class I expression, and induces successful antitumor immunotherapy. J. Exp. Med..

[134] Matsumura S., Demaria S. (2010). Up-regulation of the Pro-inflammatory Chemokine CXCL16 is a Common Response of Tumor Cells to Ionizing Radiation. Radiat. Res..

[135] Gameiro S.R., Jammeh M.L., Wattenberg M.M., Tsang K.Y., Ferrone S., Hodge J.W. (2014). Radiation-induced immunogenic modulation of tumor enhances antigen processing and calreticulin exposure, resulting in enhanced T-cell killing. Oncotarget.

[136] Liao Y., Liu S., Fu S., Wu J. (2020). HMGB1 in Radiotherapy: A Two Headed Signal Regulating Tumor Radiosensitivity and Immunity. Oncotargets Ther..

[137] Frey B., Ruckert M., Deloch L., Ruhle P.F., Derer A., Fietkau R., Gaipl U.S. (2017). Immunomodulation by ionizing radiation-impact for design of radio-immunotherapies and for treatment of inflammatory diseases. Immunol. Rev..

[138] Dolmans D., Fukumura D., Jain R.K. (2003). Photodynamic therapy for cancer. Nat. Rev. Cancer.

[139] Nakajima N., Kawashima N. (2012). A basic study on Hypericin-PDT in vitro. Photodiagnosis Photodyn. Ther..

[140] Du H.Y., Olivo M., Mahendran R., Huang Q., Shen H.M., Ong C.N., Bay B.H. (2007). Hypericin photoactivation triggers down-regulation of matrix metalloproteinase-9 expression in well-differentiated human nasopharyngeal cancer cells. Cell. Mol. Life Sci..

[141] Donohoe C., Senge M.O., Arnaut L.G., Gomes-da-Silva L.C. (2019). Cell death in photodynamic therapy: From oxidative stress to anti-tumor immunity. Biochim. Biophys. Acta Rev. Cancer.

[142] Cheng Y., Cheng H., Jiang C., Qiu X., Wang K., Huan W., Yuan A., Wu J., Hu Y. (2015). Perfluorocarbon nanoparticles enhance reactive oxygen levels and tumour growth inhibition in photodynamic therapy. Nat. Commun..

[143] Li W., Yang J., Luo L., Jiang M., Qin B., Yin H., Zhu C., Yuan X., Zhang J., Luo Z. (2019). Targeting photodynamic and photothermal therapy to the endoplasmic reticulum enhances immunogenic cancer cell death. Nat. Commun..

[144] Yang W.J., Zhang F.W., Deng H.Z., Lin L.S., Wang S., Kang F., Yu G.C., Lau J., Tian R., Zhang M.R. (2020). Smart Nanovesicle-Mediated Immunogenic Cell Death through Tumor Microenvironment Modulation for Effective Photodynamic Immunotherapy. ACS Nano.

[145] Doix B., Trempolec N., Riant O., Feron O. (2019). Low Photosensitizer Dose and Early Radiotherapy Enhance Antitumor Immune Response of Photodynamic Therapy-Based Dendritic Cell Vaccination. Front. Oncol..

[146] Chen J., Xie J., Jiang Z., Wang B., Wang Y., Hu X. (2011). Shikonin and its analogs inhibit cancer cell glycolysis by targeting tumor pyruvate kinase-M2. Oncogene.

[147] Ingemarsdotter C.K., Baird S.K., Connell C.M., Oeberg D., Hallden G., McNeish I.A. (2010). Low-dose paclitaxel synergizes with oncolytic adenoviruses via mitotic slippage and apoptosis in ovarian cancer. Oncogene.

[148] Annels N.E., Arif M., Simpson G.R., Denyer M., Moller-Levet C., Mansfield D., Butler R., Shafren D., Au G., Knowles M. (2018). Oncolytic Immunotherapy for Bladder Cancer Using Coxsackie A21 Virus. Mol. Ther. Oncolytics.

[149] Liu P., Zhao L., Kepp O., Kroemer G. (2020). Quantitation of calreticulin exposure associated with immunogenic cell death. Tumor Immunol. Immunother. Cell. Methods Pt B.

[150] Huang Y.L., Dong Y.L., Zhao J.F., Zhang L.J., Kong L., Lu J.D.J. (2019). Comparison of the effects of photon, proton and carbon-ion radiation on the ecto-calreticulin exposure in various tumor cell lines. Ann. Transl. Med..

[151] Truxova I., Kasikova L., Salek C., Hensler M., Lysak D., Holicek P., Bilkova P., Holubova M., Chen X., Mikyskova R. (2020). Calreticulin exposure on malignant blasts correlates with improved natural killer cell-mediated cytotoxicity in acute myeloid leukemia patients. Haematologica.

[152] Kasikova L., Hensler M., Truxova I., Skapa P., Laco J., Belicova L., Praznovec I., Vosahlikova S., Halaska M.J., Brtnicky T. (2019). Calreticulin exposure correlates with robust adaptive antitumor immunity and favorable prognosis in ovarian carcinoma patients. J. Immunother. Cancer.

[153] Han A., Li C., Zahed T., Wong M., Smith I., Hoedel K., Green D., Boiko A.D. (2019). Calreticulin is a Critical Cell Survival Factor in Malignant Neoplasms. PloS Biol..

[154] Tatsuno K., Yamazaki T., Hanlon D., Han P., Robinson E., Sobolev O., Yurter A., Rivera-Molina F., Arshad N., Edelson R.L. (2019). Extracorporeal photochemotherapy induces bona fide immunogenic cell death. Cell Death Dis..

[155] Hongo K., Kazama S., Tsuno N.H., Ishihara S., Sunami E., Kitayama J., Watanabe T. (2015). Immunohistochemical detection of high-mobility group box 1 correlates with resistance of preoperative chemoradiotherapy for lower rectal cancer: A retrospective study. World J. Surg. Oncol..

[156] Lea J.D., Clarke J.I., McGuire N., Antoine D.J. (2016). Redox-Dependent HMGB1 Isoforms as Pivotal Co-Ordinators of Drug-Induced Liver Injury: Mechanistic Biomarkers and Therapeutic Targets. Antioxid. Redox Signal..

[157] Dubyak G.R. (2019). Luciferase-assisted detection of extracellular ATP and ATP metabolites during immunogenic death of cancer cells. Tumor Immunol. Immunother. Mol. Methods.

[158] Vessey K.A., Ho T., Jobling A.I., Wang A.Y., Fletcher E.L. (2020). Fluorescent Labeling and Quantification of Vesicular ATP Release Using Live Cell Imaging. Purinergic Signal. Methods Protoc..

[159] Giglio P., Gagliardi M., Tumino N., Antunes F., Smaili S., Cotella D., Santoro C., Bernardini R., Mattei M., Piacentini M. (2018). PKR and GCN2 stress kinases promote an ER stress-independent eIF2 alpha phosphorylation responsible for calreticulin exposure in melanoma cells. Oncoimmunology.

[160] Geng F., Bao X., Dong L., Guo Q.Q., Guo J., Xie Y., Zhou Y., Yu B., Wu H., Wu J.X. (2020). Doxorubicin pretreatment enhances FAP alpha/survivin co-targeting DNA vaccine anti-tumor activity primarily through decreasing peripheral MDSCs in the 4T1 murine breast cancer model. Oncoimmunology.

[161] Palucka K., Banchereau J. (2012). Cancer immunotherapy via dendritic cells. Nat. Rev. Cancer.

[162] Laengle J., Stift J., Bilecz A., Wolf B., Beer A., Hegedus B., Stremitzer S., Starlinger P., Tamandl D., Pils D. (2018). DNA damage predicts prognosis and treatment response in colorectal liver metastases superior to immunogenic cell death and T cells. Theranostics.

[163] Fucikova J., Becht E., Iribarren K., Goc J., Remark R., Damotte D., Alifano M., Devi P., Biton J., Germain C. (2016). Calreticulin Expression in Human Non-Small Cell Lung Cancers Correlates with Increased Accumulation of Antitumor Immune Cells and Favorable Prognosis. Cancer Res..

[164] Fahmueller Y.N., Nagel D., Hoffmann R.T., Tatsch K., Jakobs T., Stieber P., Holdenrieder S. (2013). Immunogenic cell death biomarkers HMGB1, RAGE, and DNAse indicate response to radioembolization therapy and prognosis in colorectal cancer patients. Int. J. Cancer.

[165] Loi S., Pommey S., Haibe-Kains B., Beavis P.A., Darcy P.K., Smyth M.J., Stagg J. (2013). CD73 promotes anthracycline resistance and poor prognosis in triple negative breast cancer. Proc. Natl. Acad. Sci. USA.

[166] Ladoire S., Enot D., Andre F., Zitvogel L., Kroemer G. (2016). Immunogenic cell death-related biomarkers: Impact on the survival of breast cancer patients after adjuvant chemotherapy. Oncoimmunology.

[167] Stoetzer O.J., Fersching D.M.I., Salat C., Steinkohl O., Gabka C.J., Hamann U., Braun M., Feller A.-M., Heinemann V., Siegele B. (2013). Circulating immunogenic cell death biomarkers HMGB1 and RAGE in breast cancer patients during neoadjuvant chemotherapy. Tumor Biol..

[168] Rapoport B.L., Anderson R. (2019). Realizing the Clinical Potential of Immunogenic Cell Death in Cancer Chemotherapy and Radiotherapy. Int. J. Mol. Sci..

[169] Leon I.E., Diez P., Baran E.J., Etcheverry S.B., Fuentes M. (2017). Decoding the anticancer activity of VO-clioquinol compound: The mechanism of action and cell death pathways in human osteosarcoma cells. Metallomics.

[170] Leon I.E., Diez P., Etcheverry S.B., Fuentes M. (2016). Deciphering the effect of an oxovanadium(IV) complex with the flavonoid chrysin (VOChrys) on intracellular cell signalling pathways in an osteosarcoma cell line. Metallomics.

[171] Diederich M. (2019). Natural compound inducers of immunogenic cell death. Arch. Pharmacal Res..

[172] Juanes-Velasco P., Carabias-Sanchez J., Garcia-Valiente R., Fernandez-García J., Gongora R., Gonzalez-Gonzalez M., Fuentes M. (2018). Microarrays as Platform for Multiplex Assays in Biomarker and Drug Discovery. Rapid Test-Advances in Design, Format and Diagnostic Applications.

[173] Kepp O., Senovilla L., Vitale I., Vacchelli E., Adjemian S., Agostinis P., Apetoh L., Aranda F., Barnaba V., Bloy N. (2014). Consensus guidelines for the detection of immunogenic cell death. Oncoimmunology.

[174] Vizcaino J.A., Kubiniok P., Kovalchik K.A., Ma Q., Duquette J.D., Mongrain I., Deutsch E.W., Peters B., Sette A., Sirois I. (2020). The Human Immunopeptidome Project: A Roadmap to Predict and Treat Immune Diseases. Mol. Cell. Proteom..

[175] Juanes-Velasco P., Landeira-Viñuela A., Acebes-Fernández V., Hernández Á.P., Luque-García M., Arias-Hidalgo C., Montalvillo E., Góngora R., Fuentes M. (2021). Deciphering Human Leukocyte Antigen susceptibility maps from immunopeptidomics characterization in oncology and infections. Front. Cell. Infect. Microbiol..

[176] Exner R., Sachet M., Arnold T., Zinn-Zinnenburg M., Michlmayr A., Dubsky P., Bartsch R., Steger G., Gnant M., Bergmann M. (2016). Prognostic value of HMGB1 in early breast cancer patients under neoadjuvant chemotherapy. Cancer Med..

